# Phase-dependent stimulation response is shaped by the brain’s dynamic functional connectivity

**DOI:** 10.1162/NETN.a.548

**Published:** 2026-04-22

**Authors:** Sophie Benitez Stulz, Samy Castro, Boris Gutkin, Matthieu Gilson, Demian Battaglia

**Affiliations:** Aix-Marseille Université, INSERM, INS, Institut de Neurosciences des Systèmes, Marseille, France; Université de Strasbourg, CNRS, LNCA, Laboratoire de Neurosciences Cognitives et Adaptatives, Strasbourg, France; Université de Strasbourg, University of Strasbourg Institute for Advanced Studies (USIAS), Strasbourg, France; Ecole Normale Supérieure - PSL University, LNC INSERM DEC, Paris, France; Aix-Marseille Université, CNRS, INT, Institut de Neurosciences de la Timone, Marseille, France

**Keywords:** Dynamic functional connectivity, Brain stimulation, Phase response curve, Oscillations, Whole-brain modelling

## Abstract

External brain stimulation is a promising tool for investigating and altering cognitive processes, with potential clinical applications to the restoration of dysfunctional neural dynamics. In line with experimental observations, we study how the effects of stimulation crucially depend on the ongoing dynamics of the brain, at the local level of the stimulated region but also of global coordinated brain activity. Specifically, we use connectome-based whole-brain computational modeling to explore how the effects of single-pulse stimulation to different regions strongly depend on both the phase of regional oscillatory activity and on the transiently occurring network of functional connectivity at the time of the applied stimulation. Importantly, we show that stimulation has not only state-dependent effects but can also induce global state switching. Lastly, predicting the effect of stimulation by using machine learning shows that functional network-aware measures (i.e., knowledge of either a discrete state of functional connectivity or of a detailed functional connectivity matrix) can increase the performance by up to 40%. Our results suggest that a fine characterization of intrinsic functional connectivity dynamics is essential for improving the reliability of exogenous stimulation.

## Author Summary

Brain stimulation is a tool of growing importance to alleviate symptoms of neurological and psychiatric disorders. It is often applied in the form of local electric or magnetic pulses. Its effects, however, are nonlocal and, furthermore, dauntingly variable. Here, we construct computational models of whole-brain network dynamics and perform massive in silico experiments to identify endogenous factors influencing the effects of exogenous stimulation. Beyond previous studies that considered local oscillations, we focus here as well on the impact of global functional connectivity dynamics. We thus see that the same stimulation applied in different transient modes of functional connectivity can produce completely different effects. Thus, dynamics of large-scale functional connectivity should be taken into account to predict the effects of focal stimulation and optimize them.

## INTRODUCTION

Brain stimulation has evolved into a promising tool for investigating neurotypical processes as well as treating pathological dysfunctions (Cheng et al., 2018; Deuschl et al., 2006; Huerta & Volpe, 2009; Janssens & Sack, 2021; Rossion et al., 2015; Tremblay et al., 2019). While the range of stimulation techniques has steadily increased, they do not always reach the necessary reliability and instead exhibit large intra- and intersubject variability (Gao et al., 2022; Hartmann et al., 2016; Hinder et al., 2014; López-Alonso et al., 2014). This variability cannot be exclusively attributed to the used stimulation methodology, but is also believed to stem from the underlying endogenous dynamics of the brain (Caulfield & Brown, 2022; Julkunen et al., 2022; Li et al., 2020; Schmidt et al., 2014).

It is therefore important to identify the factors that shape stimulation outcomes in order to improve their predictability. Among these factors, stimulation phase appears particularly relevant. Several studies have shown that the phase at which stimulation is delivered influences cortico-cortical synchronization with the target region (Momi et al., 2022), the amplitude of transcranial magnetic stimulation (TMS)-evoked cortical responses (Poorganji et al., 2021), the strength of induced long-term potentiation (Zrenner et al., 2018), and behavioral performance such as reaction times and success rates in sensorimotor tasks (Peles et al., 2020). Phase also modulates tremor responses to phase-locked stimulation in Parkinsonian patients (Duchet et al., 2020). Beyond artificial stimulation, phase similarly conditions the response to sensory inputs, with baseline phase influencing perceptual and neural outcomes independently of stimulus features (Gransier et al., 2021; Ten Oever et al., 2020; VanRullen et al., 2011).

Given the prominence of oscillatory epochs in brain activity time series, such phase dependence is far from surprising. Dynamical systems theory has long anticipated phase-dependent responses as a natural property of coupled oscillators, including interacting neural populations (Dumont & Gutkin, 2019; Kuramoto, 1991). Moreover, such sensitivity to stimulation phase may be further amplified when brain networks operate in a near-critical regime (Avramiea et al., 2020).

Phase of local oscillatory activity, however, is not alone in determining the stimulation outcomes in such experimental protocols. Transient activity states (e.g., EEG microstates) have been shown to also modulate the effect of stimulation (Croce, Zappasodi, Spadone, et al., 2018), and, in turn, stimulation may modify these dynamical network state at various spatio-temporal scales (Croce, Zappasodi, & Capotosto, 2018; Gold et al., 2022; Shang et al., 2020; Witt et al., 2013). Beyond microstates, it is known that whole-brain-level coordinated dynamics displays prominent fluctuations, with a strong degree of spatio-temporal organization at rest (Fox & Raichle 2007) and persisting also during active cognition (Cole et al., 2014). Such intrinsic structured variability gives rise to a dynamic functional connectivity (dFC; see Hutchison et al., 2013, or Calhoun et al., 2014, for a review), switching between alternative functional connectivity (FC) states and flowing across transient meta-stable configurations (Allen et al., 2014; Arbabyazd et al., 2020). Previous modeling studies have shown that the distributed effects of stimulation—such as spectral changes in regions remote from the stimulation site—are better predicted by functional rather than structural connectivity. In particular, the response at a distal site scales with its path distance from the stimulation target, but along FC paths rather than anatomical ones (Papadopoulos et al., 2020). In light of this prediction, we anticipate that the very same stimulation, when applied during different transient FC states, may give rise to distinct patterns of distributed effects. It is thus important to quantify the impact of FC state switching and to disentangle them from the influence of local regional dynamics.

To understand how transient local and global dynamics interacts with exogenous stimulation without being limited by the noisiness and the scarcity of empirical data, we perform massive in silico stimulation experiments using a large-scale connectome-based brain model displaying complex oscillatory activity (similarly to Papadopoulos et al., 2020). We thus find that stimulation is both phase- and FC state-dependent and that the exact phase-sensitivity of stimulation depends on the current dynamical network state. To separate the respective phase and state-dependent contributions, we contrast the notions of *state switching* (global reorganization of functional networks) against *state morphing* (local adjustment of a few FC links without overall network state change). Specifically, the probabilities to obtain both morphing and switching for a given stimulation configuration are phase-, FC state-, and region-dependent. Predicting stimulation effects requires therefore a complex mathematical mapping capable of jointly accounting for multiple interacting factors, including phase, state, and region.

In such a multifactorial context, a machine-learning approach becomes a viable alternative to formal theoretical models, which may depend on assumptions that are too strong to be reliable or may simply be intractable due to the system’s complexity and heterogeneity. A mapping can instead be learned between stimulation features and system-state descriptors on one side, and the resulting phase shifts on the other, yielding predictions of varying accuracy depending on the chosen input features. Here, we find that, relative to machine-learning predictions based solely on stimulation features and the local activity of the stimulated region, incorporating features that describe the current system-wide FC enhances prediction accuracy by more than 40%.

These results suggest thus that improving the reliability of stimulation requires monitoring transient whole-brain network dynamics, rather than relying exclusively on the phase of activity within the targeted region.

## RESULTS

### Could the Phase Response Be State-Dependent?

**Figure 1.**
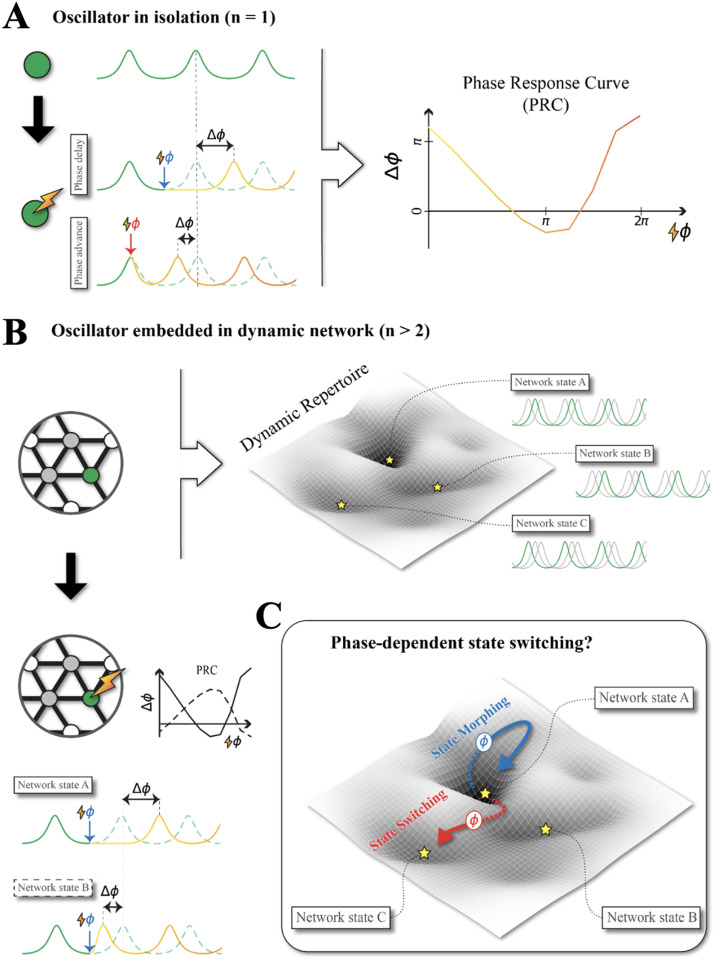
The response of a region to stimulation could depend on local and global oscillatory dynamics. Applying a precisely timed pulse of stimulation to a brain region displaying oscillatory activity may advance or delay the phase of the local oscillation (positive or negative phase shift). However, the effects of the stimulation may depend on a variety of dynamic factors. (A) The effects of stimulation pulses may depend on the phase of the local oscillation in the target region at which they are applied (left; dashed line, unperturbed oscillations; blue or red arrows, phased stimulations inducing phase advance or delay). This phase-dependency of phase-shifting effects is quantified by the PRC (see cartoon curve at the right), describing the induced phase shift as a function of stimulation phase. (B) In complex brain networks involving multiple interacting brain regions, there could be multiple system’s level oscillatory configurations characterized by different levels and lags of inter-regional phase locking, that is, alternative FC states, here represented as attracting valleys in an idealized landscape (top). In each of these states, an embedded oscillating regions would receive different inputs from the same neighboring regions, as a function of the currently visited FC state. As a net effect of the intrinsic node PRC and of these different network influences, the responses to phased stimulation pulses could become FC state-dependent (bottom left), as it could be described conveniently by introducing different effective PRCs for each state (in the cartoon, solid and dashed curves represent different effective PRCs for two distinct FC states). (C) Furthermore, the applied perturbation could induce: a limited reconfiguration of a few phase-locking links, without altering the overall geometry of the FC network and thus without leaving the current FC state (blue path, “state morphing”); but could also trigger a transition to a different FC state (red path on the landscape, “state switching”) leading to more widespread network reconfigurations. To measure state-dependent effective PRCs, the joint effects of state morphing and potential state switching must be disentangled.

In the physics of coupled oscillating system, the response to a precisely phased external perturbation is described in terms of the effects it induces on the phase and the amplitude of the stimulated network node’s oscillations. Both these effects are generally dependent on the phase of the applied perturbation, as captured by suitable phase and amplitude response curves. Amplitude response is often ignored as a first approximation and one focuses thus on the phase response curve (PRC), which describes the phase shift of an oscillator as a function of the phase at which stimulation is applied (Kuramoto, 1991). In Figure 1A, we show an example cartoon of a PRC, such that stimuli of identical intensity and duration may advance (or delay) the phase of the oscillator, depending on the positive (or negative) sign of the PRC at the stimulation phase.

The PRC is an intrinsic property of each oscillating node, which can also be used to predict possible phase-locking patterns between weakly coupled oscillators (Pikovsky et al., 2001) and even capture the effects of stimulation on their coupling (Witt et al., 2013). However, large-scale (whole-brain) networks with a multitude of oscillatory regions usually exhibit a very rich dynamic repertoire with a multiplicity of global dynamical states (Cabral et al., 2017), characterized by different degrees of inter-regional phase-locking and alternative patterns of phase lag. Each of these oscillatory states correspond to different FC configurations (Battaglia et al., 2012; Hansen et al., 2015; Kirst et al., 2016). When applying an external perturbation to a region in such a complex network, the effect on the stimulated node will also depend on the ongoing activity in the other coupled node, in a way which could become quickly unpractical to predict in terms of the “real” PRC of the node itself. It may thus be convenient to describe the stimulation response of a node embedded in a network in terms of an “effective PRC,” describing the net phase-shifting effects of an applied phased perturbation, as a combined influence of both the intrinsic phase response of the stimulated node and the influences from the neighboring nodes ongoing oscillations. Whenever multiple FC states existed, then this effective PRC would become state-dependent, as the same region would receive different phase-dependent inputs from its network neighborhood depending on the current FC.

In Figure 1B we show a cartoon illustrating the possible existence of a state-dependent effective PRC, where the same perturbation applied at the same phase would induce a phase advance in one FC state but a phase delay in another. Although the “real” PRC remains the same in both states and the different effects are due to the different phase relations with network neighbors in different states, this state-dependent diversity of effects can be summarized by introducing two distinct effective PRCs (solid and dashed lines) for the two FC states. In the following, we will have to perform phased stimulation experiments in different FC states of the model in order to measure state-dependent phase-shifting effects and extract the different effective PRCs.

### State Morphing or State Switching?

In Figure 1C, we represent the possible dynamical configurations of the network system as an idealized landscape, in which the minima of different valleys would correspond to alternative FC states prescribing different phase-locking patterns. The effective PRC of each of these states would thus describe the observed phase-dependent phase-shifting effects of a phased stimulation, supposing that the FC state remains stable (and, with it, the phases of incoming inputs from network neighborhood), that is, that the system does not leave the landscape valley which it was in at stimulation onset (blue path in Figure 1C). The stimulation in this case does not modify the system’s state but just shifts the phase of the stimulated region, slightly modifying some selected links of the current FC adjacency matrix. For this reason, we speak of “state morphing” (cf. Papadopoulos 2021). The effective PRC of a state can then be considered to describe which are the stimulation phases at which the strongest state morphing effects can be obtained.

There is, however, another possibility. In some cases, the transient effects induced by the stimulation could be such to cause the system to leave the valley in which it was to enter instead a second, different valley (red path in Figure 1C). This situation would correspond to a situation of *state switching*, in which the FC network would undergo a more global and widespread reconfiguration, due only in part to the stimulation but also especially to endogenous forces produced by the system itself self-organizing into a different FC state (Battaglia et al., 2012; Kirst et al., 2016; Witt et al., 2013). The possibility of state switching makes the estimation of FC state-specific effective PRCs more difficult. Indeed, the phase-shifting effects observed in a stimulation experiment could reflect state-switching effects, that should be disentangled from state morphing to properly measure state-specific effective PRCs. Note that the probability of state switching could itself be phase-dependent (cf. Battaglia et al., 2012; Witt et al., 2013). In the following, we will thus have to devise techniques to determine in which FC state the stimulation is performed and in which FC state the system is landing after the stimulation.

To remove the disturbance of possible confounding factors usually present in experimental data from methodological or behavioral sources, we will focus on simulations of a computational model of whole-brain dynamics. As we will show, the emergent dynamic complexity of this idealized, noise-free deterministic model will be already sufficient to enforce a large variety of state morphing and switching behaviors while making them easier to track and detect.

### Computational Model of Whole-Brain Network Dynamics

**Figure 2.**
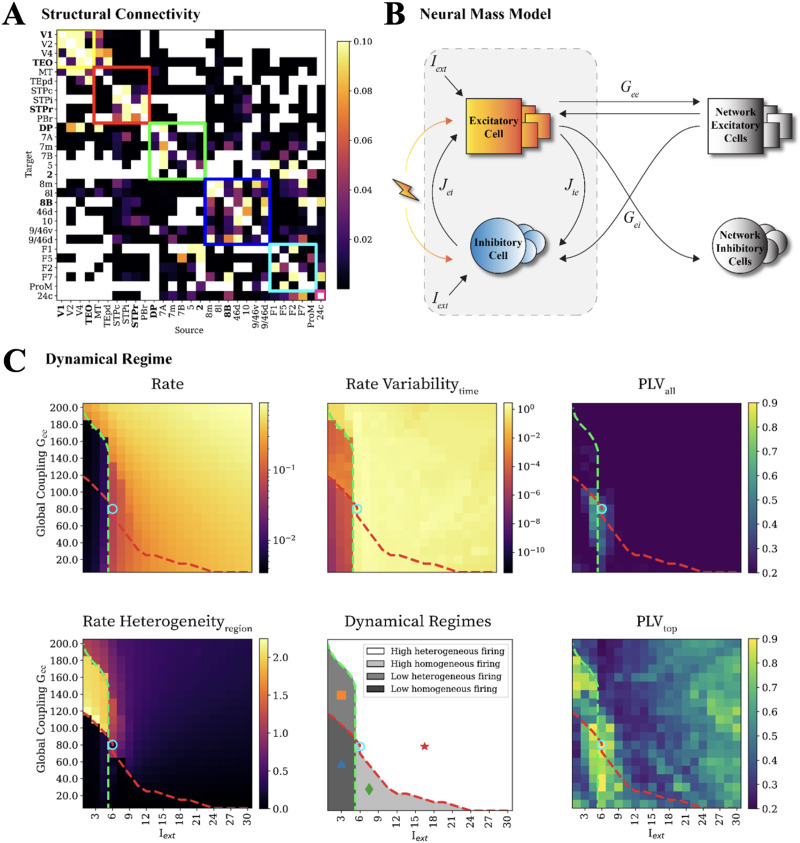
Connectome-based model of whole-brain oscillatory dynamics. We construct a computational model of resting-state brain activity to study the effects of local and global dynamics on the effects of phased stimulation. The model embeds (A) a structural connectivity matrix linking 29 brain regions, derived from empirical tractography experiments (in nonhuman primates, Markov et al., 2014). (B) Each regional neural mass consists of E and I populations that receive a background excitatory drive *I*_*ext*_. Local regional parameters are set to give rise to stable local oscillations. The relative influence of inter-regional inputs and local dynamics is tuned by a coupling parameter *G*_*ee*_ that scales the weighed and directed connectome. Long-range disynaptic inhibition *G*_*ei*_ is also present and scaled proportionally to *G*_*ee*_. (C) The dynamical regime of the whole-brain model is set by adjusting the global parameters *G*_*ee*_ and *I*_*ext*_, whose changes modify: the average rate of activity (top left); the variability in time of this rate (tracking oscillatory amplitude, top middle); the heterogeneity in firing rate across regions (bottom left). Variations in these four observables span four possible regimes with lower or higher firing rate and lower or higher homogeneity of firing across regions (see cartoon phase diagram, bottom middle). Changes in parameters affect also FC and its dynamics. We quantify FC in terms of pairwise inter-regional phase locking value (PLV) tracking the consistency in time of phase relations. PLV_*all*_ corresponds to the average over all FC links (top right) and PLV_*top*_ to the average over the top 25% strongest FC links (bottom right). We select in the following a working point (blue circle) at the crossing between multiple critical lines, maximizing simultaneously PLV_*all*_ and PLV_*top*_ and thus corresponding to the global dynamical regime with the strongest and most widespread inter-regional synchronization.

To generate rich oscillatory dynamics at the whole-brain level, we combine a weighted and directed structural connectivity derived from macaque tractography (Figure 2A) with a neural mass model consisting of coupled (mean-field) excitatory (E) and inhibitory (I) populations (Figure 2B).

We choose to use a structural connectome matrix derived from Markov et al. (2014) because of several reasons. Firstly, it is derived from tracer injections rather than MRI tractography more often used in virtual brain models (Deco et al., 2011). The reconstructed connectome matrix is directed, asymmetric, and dense, unlike MRI-diffusion-based matrices. Both features are expected to boost dynamic complexity, through symmetry breaking (Pillai & Jirsa, 2017). Secondly, this ground-truth connectivity information involves a moderate number of regions, which provides an interesting balance between complexity and feasibility for our computationally intensive in silico experiments.

To generate plausible oscillatory dynamics within each region, the E/I populations are coupled in a Pyramidal-Interneuron Network Gamma (PING)-inspired fashion (Figure 2B). Note that this mean-field model for each population is an exact reduction of spiking activity, so the population dynamics can be traced back to neuronal and synaptic parameters (Montbrió et al., 2015). Similar to the PING configuration, which is an established canonical description of cortical oscillations in the gamma range (Whittington et al., 2000), our neural mass model gives rise to a biphasic PRC (Dumont & Gutkin, 2019; Montbrió et al., 2015). Analogous to the one of Figure 1A, stimulation can induce both phase advancements and delays when stimulating simultaneously both E and I populations (simulating cell-type aspecific empirical stimulation).

Before studying stimulation responses, we explore the effects of global excitatory-to-excitatory coupling Gee and the local background excitatory drive Iext (same for all regions) on the overall network dynamics. In this way, the connectome matrix sets the relative strengths of different inter-regional excitatory connections, but not their absolute strengths (scaled by the parameter Gee). Inter-regional connections are purely excitatory and target both the E and I populations, with a fixed ratio between Gee and Gei conductance (see Material and Methods section; Figure 2B). Together, the two parameters Gee and Iext determine the regime of collective dynamics, which we characterized using various metrics in Figure 2C and Supporting Information Figure S1.

Simulating time series in a resting state (i.e., constant background inputs without stimulation), we analyze the firing rate across time and regions. The average firing rate (Figure 2C, top left) quantifies the overall activity of the network. The rate variability across time corresponds to the firing fluctuations over time (normalized and averaged over regions) as shown in Figure 2C (top, middle), which reflects the oscillation amplitudes. The rate heterogeneity of time-averaged rate across regions is shown in Figure 2C (bottom, left), with high values for regimes where only some regions are strongly active while others are quiescent. Finally, since the number of strongly activated regions may change over time, we also compute the temporal variability of the spatial rate heterogeneity (Supporting Information Figure S1, top right). See Materials and Methods section for precise definitions of these four collective dynamics descriptive features.

Pooling the observed trends, we find four qualitatively distinct dynamical regimes, indicated by different symbols and colors in the summary phase diagram in Figure 2C (bottom, middle), and separated by two crossing dashed lines. The regimes left of the green vertical line (lower values of Iext) have low firing rates, as well as oscillations, as compared to those on the right. The regimes under the red diagonal line display homogeneous firing rates for all regions, whereas regional heterogeneities increases above it (especially for high values of Gee and low values of Iext). Therefore, we have the following four distinct regimes: with homogeneously low (dark gray, triangle) or high (light grey, square) firing rates; and with heterogeneously low (intermediate grey, rhombus) or high (white, star) firing rates. See Supporting Information S1 for example time series for each regime from the working points (WP) indicated by each symbol.

Beside the oscillatory firing rates, we also choose the WP by characterizing the synchronicity of oscillatory dynamics using the inter-regional PLV, which captures the existence of temporally stable phase differences between pairs of regions (Lachaux et al., 1999). The PLV averaged over all pairs of regions is generally very low, as indicated by dark blue color in Figure 2C (top right). Interestingly, however, in proximity of the crossing of the red and green dashed lines defining the transitions between firing-rate based regimes, the average PLV peaks (reaching ~0.75). This means that the critical zone in proximity of the convergence between regime boundaries is associated with enhanced global phase locking. In the following, we will perform stimulation experiments at the WP at the cusp between regimes (Gee = 80.0; Iext = 6.0), where high global synchrony could facilitate (at least in principle) the detection of stable and reliable phase-dependency effects. We further examine whether subnetworks of regions are highly phase locked. This more “local” synchrony in the network can be detected by calculating the average PLV of the 25% of links among all possible region pairs (Figure 2C, bottom right). This analysis reveals that synchronized subsets of regions can exist even far away from the critical line crossing point. Remarkably, however, even in the critical zone, the average top 25% of most synchronized pairs of regions reaches values of up to ~0.89, well above the global average. This finding indicates that the degree of phase-locking is heterogeneous and that there could be cores of highly synchronized regions, raising the question of their stability or transiency over time. Eventually, fixing the model’s global parameters to select a specific dynamic working point does not preclude the dynamics from exploring a multiplicity of transient metastable configurations within that regime.

### Transient FC State Extraction

In order to evaluate and predict the effect of stimulation, our underlying assumption is that the transient dynamic network state must be incorporated into the analysis. This requires the quantitative characterization and extraction of such states from the collective network activity. We decided to rely on FC, which captures the coordination between pairs of regions, rather than region-specific dynamical properties. FC is thus particularly suitable for tracking system’s level dynamical configurations (Kirst et al., 2016) and their evolution in time (Hutchison et al., 2013). Here, because of the oscillatory nature of the considered dynamics, we define FC in terms of oscillatory phase locking (quantified by PLV between all pairs of regions) and inter-regional phase differences quantified by normalized phase lag (e.g., equal to 0 for in-phase locking and to 0.5 for anti-phase locking, see Materials and Methods section). The rationale is to capture not only the presence of synchronization, but also information about the extent to which regions precede each other, as we detail now.

**Figure 3.**
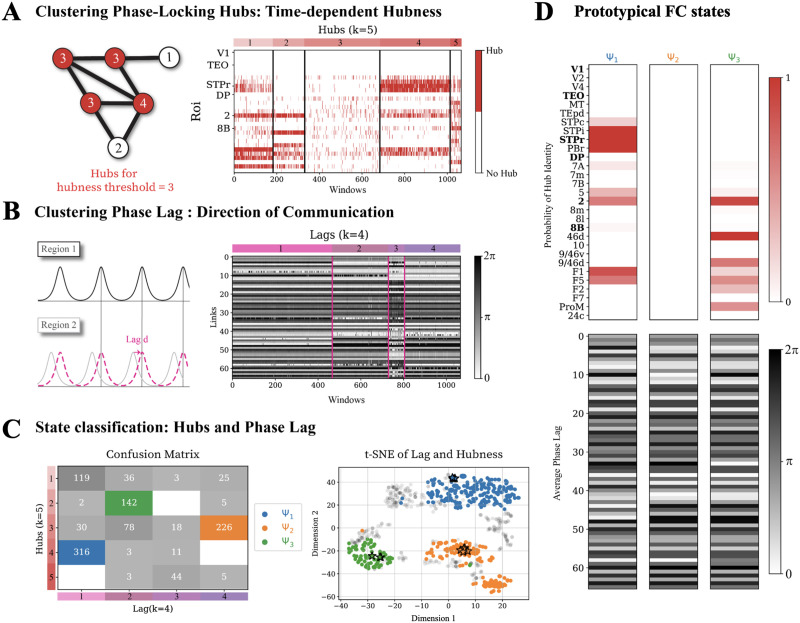
Extraction of FC states. An FC state is characterized by the set of regions that are “hubs” for this specific state (high strength in the FC adjacency matrix) and by the set of inter-regional phase lags of pairwise PLV links. Analyzing long simulations of resting-state activity at the chosen working point (see Figure 2C), we quantify instantaneous FC strengths and phase lags and try to group different epochs into a few discrete homogeneous clusters, corresponding to putative FC states. (A) Time-dependent hubs are defined as nodes with high degree in a transient FC network (links binarized via thresholding on PLV values, see Materials and Methods section). Left: cartoon representing hubs (degree larger than 3 in this example). Right: hubs identified for different transient FC networks. Rows denote different regions (hubs are marked in red color) and columns different time frames. (B) Left: cartoon describing phase lag between the time series of two oscillating regions. Right: vectors of phase lags for different transient FC networks. Columns correspond once again to time frames and rows to different links of pairwise FC. In panels B (or C), time frames have been regrouped in four (or five) clusters, obtained by similarity of their hubness (or phase lag) vectors in the associated transient FC networks. (C) As visible from the confusion matrix between hubness- and lag-based clusters, at least three states with consistent hubs and phase lags are relatively more common than others in our reference simulations. We refer to them in the following with the arbitrary labels Ψ_1_, Ψ_2_, and Ψ_3_. Left: dimensionally reduced projection (via the t-SNE distance preserving algorithm) of the instantaneous concatenated hubness and lag vectors of the transient FCs at different times. Each dot corresponds to the two-dimensional projection of an instantaneous PLV-based FC network. Dots belonging to the three states of interest Ψ_1_, Ψ_2_, and Ψ_3_ are correspondingly color coded, while instantaneous FC out of these three most frequent states are shaded in gray. Stars indicate prototypical instances of FC for each state, whose hubness and phase-lag vectors are represented in panel D.

To measure FC in a dynamic fashion, we use a sliding window approach (window length = 140 timesteps, overlap = 75%) and then extract our FC measures for each (time) window. For each window and each region, we calculate a “hubness” measure, which is larger (smaller) for strongly phase-locked regions with many (few) other regions (Figure 3A, left). We call regions, whose hubness exceeds a chosen threshold, phase-locking hubs. Since the PLV matrices change over time (i.e., across windows), we stress that hubness and hub identity are dynamic properties: different regions can be labeled as phase-locking hubs at different times. This is shown in Figure 3A (right), where red bars denote regions labelled as hubs in a given window. The windows have been reordered according to an unsupervised clustering (in *k* = 5 cluster) of the time-dependent sets of hubs (see Materials and Methods section and Supporting Information Figure S2 for details of hubness analyses), yielding five groups over all considered windows.

The normalized phase lag (or, in short, lag; cf. Supporting Information Figure S3.2) gives a proxy for the direction of communication based on cross-correlation (Palmigiano et al., 2017). Once again, lags are dynamic and the same FC link with strong phase locking can exhibit different preferred phase differences across windows (Figure 3B). Vectors of phase lags in Figure 3B have been reordered in four groups, once again based on unsupervised clustering.

Interestingly, these groups extracted from hubness on the one hand and lag on the other largely overlap as shown by the confusion matrix in Figure 3C (left). This identifies the presence of at least three distinct clusters with well-defined sets of hubs and inter-regional phase relations. These clusters are also easily visible as disconnected clouds (each dot representing a window) in a joint dimensionally-reduced representation of hub sets and phase lags (Figure 3C, right), obtained by t-distributed stochastic neighbor embedding (t-SNE; van der Maaten & Hinton, 2008). We refer to these three clusters as states Ψ_1_ (blue), Ψ_2_ (orange), and Ψ_3_ (green). Figure 3D shows a graphical representation of their centroids, corresponding to stereotypical patterns of PLV-based hubs and lags. Note that these states are not the only patterns exhibited by our dynamic system and we group all remaining patterns under a generic fourth group “None,” beside the well-defined states Ψ_1_, Ψ_2_, and Ψ_3_.

Through this double-clustering procedure, every window is attributed to a state label. Inspecting the sequence of state labels over time, we find that states are temporally stable: at each time, it is more probable to remain in the same state in the next window, than to switch to another state (cf. Supporting Information S3); in fact, transitions between states occur via windows from the “None” group. This temporal stability allows us to isolate time epochs in which the system consistently visits a given state, such that the application of stimulation in such epochs enables the study of the state-dependency of its effects.

#### Simulated phase-shifting effects depend on stimulation phase, FC state, and region.

In order to examine the state-dependent effects of stimulation, we simulate a single pulse stimulation to a single selected region applied within windows corresponding to one of the FC states (Ψ_1_, Ψ_2, _ or Ψ_3_). The spread of the state clouds indicates some variability across windows within each state (Figure 3C, right; see Discussion and last section in Results); therefore, we constrain our study to highly representative windows for each FC state to capture state-specific responses. We thus select five typical windows that are most similar to the centroid of each state cloud, while being far from other state clouds (see stars in Figure 3C, right). Within those selected windows, we perform multiple stimulation experiments, applying individual stimulation pulses at different phases in each simulation (see Material and Methods section for details on stimulation simulation). Note that we select stimulation points within long-lasting epochs within a stable state so that the observed phase-shifting effects can be attributed to the stimulation rather than to spontaneous variability (we compare indeed stimulated system’s trajectories with unstimulated ones, using precisely the same initial conditions and stochastic parameters realizations, see Materials and Methods section).

The numerous dimensions explored here (phase, FC state, regions) increase the computational cost of numerical simulation exponentially. Concretely, we chose to explore 10 different phase bins and three different FC states with 25 samples (five different oscillatory period cycles in each of the five different typical windows) for each combination such as to average over multiple instances to quantitatively capture the variability of stimulation effects; this choice yields 750 simulations for each stimulated region. An exhaustive study of all 29 regions would thus require running 21,000 individual simulations of the dynamics of a whole virtual brain modulated by stimulation. These numbers put a limit on the practical feasibility of exhaustively investigating all combinations.

Therefore, we focus on a subset of six regions from various cortical areas and different levels of phase locking within the network: TEO in the visual area, STPr in the motor area, DP in the parietal area, and 8B in the prefrontal area (see bold names in Figure 2A. Furthermore, we also added the region with the fewest hubs (V1) and the region with the) most hubs (2) thereby covering a variety of regions. Investigating stimulation effects over this subset of regions still required running over 4,000 in silico stimulation experiments, which is a more realistic, yet daunting, endeavor.

To capture the effect of each stimulation pulse, we simulate two sets of (multivariate) time series of whole-brain activity in parallel: the unstimulated and the stimulated time-series that only differ in terms of whether stimulation was applied at a specific phase, region, and FC state. Because our model is deterministic and the parallel simulations start from the same initial conditions, we can be sure that the observed deviations are exclusively due to the stimulation. Furthermore, this determinism allows us to anticipate the phase at which we apply the stimulation with complete precision (an advantage compared to empirical experiments). Stimulation intensity was chosen pragmatically so as to avoid amplitudes that were too strong—which produced exaggerated effects largely independent of the stimulation parameters—and amplitudes that were too weak, which yielded inconsistent and mostly nonsignificant effects (see Supporting Information Figure S4 and Materials and Methods section).

**Figure 4.**
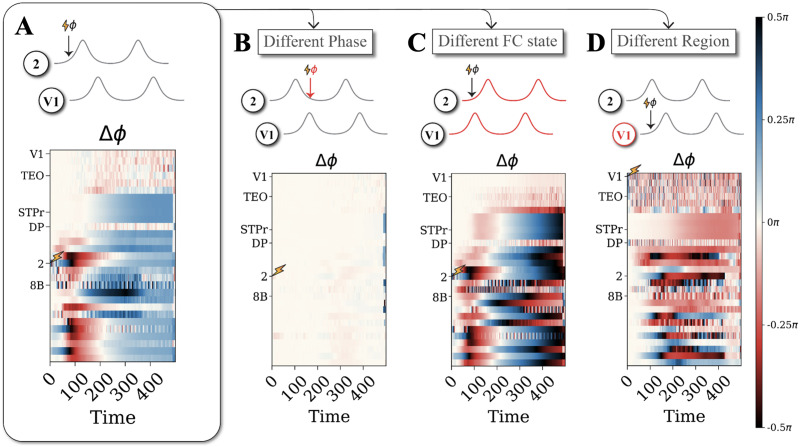
Stimulation effects depend on phase, FC state, and region. We show here phase shifting portraits, that is, the phase shift with respect to an unstimulated case (Δ*ϕ*) induced by a phase single-region stimulation as a function of poststimulation time and region. Phase-shifting portraits differ strongly across each dimension of interest: phase, FC state, and region of stimulation. (A) Phase-shifting portrait of an example phased stimulation to a reference region (region 2) at a reference stimulation phase (0.4*π* radians) in a reference FC state (Ψ_1_). Phase-shifting portraits are completely different when fixing all stimulation parameters but changing either: phase of stimulation (A, now 1.2*π* radians); FC state of stimulation (B, now Ψ_2_); or region of stimulation (C, now region V1).

To calculate the phase-shifting effect of the stimulation, we subtract the phase of unstimulated time series from the phase of the stimulated time series. We stress that we evaluate the effects after averaging over 25 repetitions for different oscillation cycles, but with the same initial conditions in terms of region, state, and phase where the stimulation is applied (see Materials and Methods section). The phase response goes through a transient shifting behavior initially at ~100 timesteps and stabilizes after ~400 timesteps (see Figure 4A). In line with previous studies (Gold et al., 2022; Momi et al., 2022; Momi et al., 2023; Ozdemir et al., 2020), the average phase-shifting effect of stimulation is also highly nonlocal (i.e. regions far away the stimulated one can be affected; cf. Papadopoulos et al., 2020). Furthermore, it is heterogeneous across affected regions meaning that there is a structured network-level response (see Figure 4A).

In line with the concept of the PRC, stimulating at a different phase with otherwise identical parameters can lead to strikingly different patterns of phase shifting across regions (cf. Figure 4A and B). While there is a strong network response in 4A for a phase of 0.4*π*, the response is flat in 4B when stimulating at phase 1.2π for exactly the same region and same initial condition within the same FC state. This confirms the presence of phase-dependent effects in our simulation experiment.

Most importantly, we also see strongly divergent patterns when we stimulate in a different FC state (cf. Figure 4A and C), which is a key prediction of our main hypothesis. While both phase-shifting time series display similar initial transients at ~100 timesteps, the phase-shifting trajectories diverge at a later stage. Indeed, after equilibration at ~400 timesteps, there is a global phase advancement for Ψ_1_ in 4A, whereas there is a heterogeneously structured phase delay for Ψ_2_ in 4C.

Lastly, stimulation effects also depend on the region of application as network-wide phase-shifting profiles change when different regions are stimulated (Figures 4A and 4D). For example, an initial transient response emerges around ~250 timesteps and not ~100 timesteps when stimulating region V1 (Figure 4D) and settles as a global structured phase delay and not an advance after 400 timesteps. Some regions such as 8B, DP, and STPr were extremely rigid in response to stimulation for all FC states and phases (Supporting Information S5), whereas other regions were sensitive during specific FC states (V1) or during specific phases (2). Region TEO has some weak stimulation effects while regions such as 8B show phase-independent effects in Ψ_2_ or regions such as V1 demonstrate both phase- and FC-state-dependence. These results emphasize that the stimulation responses in a complex oscillatory network are multifactorial and cannot be explained by a single factor.

### Disentangling State Switching From State Morphing

A possible explanation underlying some of the heterogeneous phase shifting profiles shown in Figure 4 could be that stimulation in different conditions may induce different phenomena. Indeed, phase shifting due to induced state morphing (Figure 1B) may be more moderate and more local, while phase shifting due to induced state switching (Figure 1C) is expected to be more radical and distributed across the whole network. We thus track the state labels for the parallel stimulated and unstimulated time series in windows following the stimulation to properly discriminate between the state morphing and switching cases. This is possible in our in silico experiments by means of a trained machine-learning classifier: we rely on the state labels obtained via the unsupervised clustering of resting state (unstimulated) dynamic FC matrices (cf. Figure 3) and use them to generalize the classification to those engendered by stimulated times series. The classifier allows us to assign a state to which the transiently observed PLV and lag configuration most likely belongs to (see Materials and Methods section). Stimulated trials are then sorted into two groups: trials starting and ending in the same state (phase shifting effects corresponding to state morphing) and trials with a different end state compared to the start state (hence, state switching). We stress that disentangling state morphing from state switching cannot be achieved through a simple metric, because the difference between the two is qualitative rather than quantitative: individual phase differences may be of comparable magnitude in both cases. Instead, identifying the distinction requires a global inspection of the correlations among distributed phase-shifting effects across multiple links simultaneously—a task that is feasible through our machine-learning procedure but not reducible to a simple analytical formula.

Recall also that this enforced discretization with three FC states is an approximation that neglects the variability within each FC state, which can result in high variability across stimulation effects with the same conditions on phase, state, and region (cf. Supporting Information Figure S5). We will discuss later this “limitation” of our description (see also *Discussion*), but we focus on the distinction between morphing and switching in the following.

**Figure 5.**
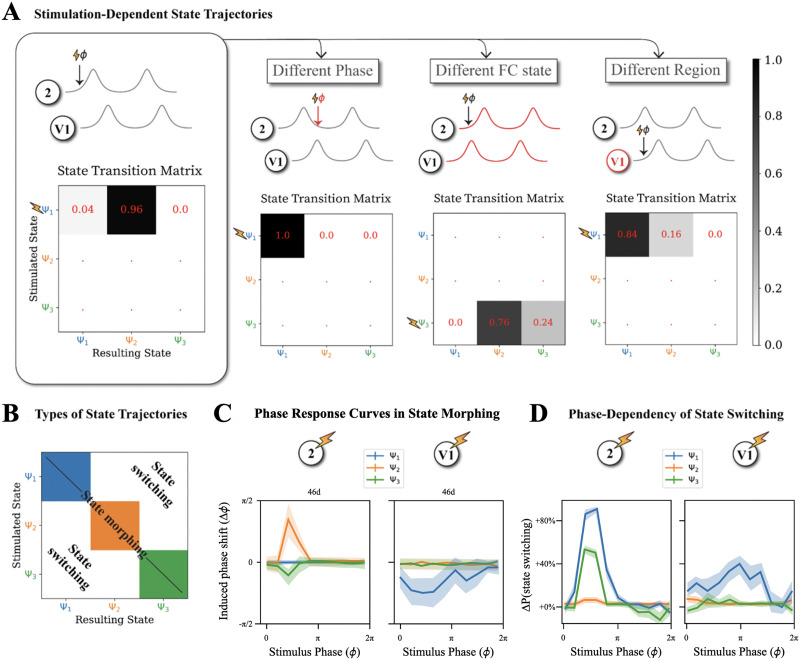
Stimulation can induce both state morphing and state switching. To disentangle state morphing and switching, we need to know the FC state of the system both before and after stimulation, which is possible by constructing a machine-learning classifier learning to assign a FC state label to previously unobserved FC frames. (A) Similarly to phase-shifting portraits in Figure 2, the probabilities of inducing a switching from one state to another depend on the phase, the starting FC state and the region of stimulation. (B) We filter individual stimulation trials depending on them: not inducing state switching (probabilities given by diagonal entries of the state transition matrix) and thus suitable for evaluating FC-state-specific effective PRC; or inducing state switching (off-diagonal entries of state transition matrix) and thus suitable for evaluating the phase dependency of state switching probability. (C) Example effective PRCs of a representative region (here 46d) describing state morphing within different FC states for stimulation of two different regions. (D) Probabilities of a phased perturbation inducing state switching as a function of the phase of stimulation, of the starting state and of two example stimulated regions. We plot specifically the excess—or defect—probability of state switching ∆P, obtained as a net difference with the spontaneously expected rate of switching between states as an effect of endogenous resting state fluctuations, hence the positive—or negative—values of the points on these curves. In panels C and D, shaded intervals indicate standard deviation of the mean.

Considering state switching, we consider the same stimulation experiments as in Figure 4 and quantify the probability of stimulation-induced state switching, rather than a generic mixed-origin phase shifting. We find that state switching occurs consistently, for the example in Figure 4A, from state Ψ_1_ to Ψ_2_ in 96% of the cases (Figure 5A, first column) and, for the example in Figure 4C, from state Ψ_3_ to Ψ_2_ in 100% of the cases (Figure 5A, third column). However, state switching does not occur for the examples shown in 4B and 4D (Figure 5A, second and fourth columns, respectively). This confirms that the probability of state switching also depends on phase, state, and region as in Figure 4.

#### State morphing is shaped by the dynamic FC state.

Now focusing on stimulated trials without state switching, we study the weaker phase shifting effects associated with state morphing. To discard transient effects, we measure stimulation-induced phase shifting in a late poststimulation window (360–500 timesteps after stimulation) and compile these measurements into PRCs describing their phase dependency, state by state. Even after excluding all trials with state switching, the extracted PRCs are strongly affected by FC states (Figure 5C). For instance, when region 2 is stimulated in FC state Ψ_1_ and Ψ_3, _ its phase is unaffected; however, when stimulating it in FC state Ψ_2_, we observe consistent phase delays for stimulation phases around ~*π*/2 (Figure 5C, center left). Expectedly, the PRCs depends on both the stimulated region and the affected region as shown for state Ψ_2_ with phase advancements versus delays (Figure 5C). While the PRCs for STPr, 8B, DP, and 2 show very consistent responses across samples, those for TEO and V1 are dominated by variability of larger magnitude than the average phase shift (see Supporting Information S7) due to large single trial variability (Supporting Information Figure S6). This observation indicates once again (cf. Supporting Information Figure S6) that description of transient activity can be refined by going beyond the defined discrete states.

#### State switching is dependent on the phase of stimulation.

Following Figure 5A with dependency of state switching upon phase, FC state, and region, we further represent this information in terms of curves for the phase-dependent probability difference of switching (Figure 5D), which is more directly comparable with PRCs associated with (state-dependent) state morphing (Figure 5C).

Interestingly, regions with a high probability of stimulation-induced state switching such as region 2 (and V1) also show a strong phase and FC state dependence in their state switching probabilities (see Figure 5D, left; Supporting Information S8). For example, in region 2, states Ψ_1_ and Ψ_3_ are very likely to switch to another state when the stimulation arrives at phase ~*π*/2, whereas Ψ_2_ is much more robust and its switching deviation is uniformly low for all phases and stimulation even seems to suppress natural state switching (for negative values). In regions such as 8B (or DP, TEO, and STPr) for which the propensity of stimulation-induced state switching is lower, there was also no strong phase dependence (see Figure 5D, right; Supporting Information S8). Overall, strong state switching deviations can be induced by applying stimulation in specific combinations of phase windows and regions, as previously observed by other studies (Battaglia et al., 2012; Witt et al., 2013).

Clearly, FC states are an important dimension to consider when attempting to understand the effect of stimulation in a complex oscillatory network. The results in Figure 5C–D show the limits of the traditional PRC concept and how the effects of stimulation transcend it. On the other hand, it may quickly become impractical to build a dictionary of all possible state dependent PRCs in large networks. In order to understand and predict stimulation effects, we thus need to introduce more straightforward strategies, explicitly accounting for the existence of complex dependencies for multiple factors simultaneously.

#### State-aware information is essential for predicting stimulation-dependent phase shifting.

To condense the rich multidimensional information contained in our simulated stimulation experiments and make practical predictions on the resulting effects, we rely on machine-learning tools. Specifically, we train a Random Forests Regression algorithm (see Materials and Methods section) to predict the stimulation-induced phase shifts using cross-validation based on a flexible combination of input variables, which describe the network with various levels of granularity.

**Figure 6.**
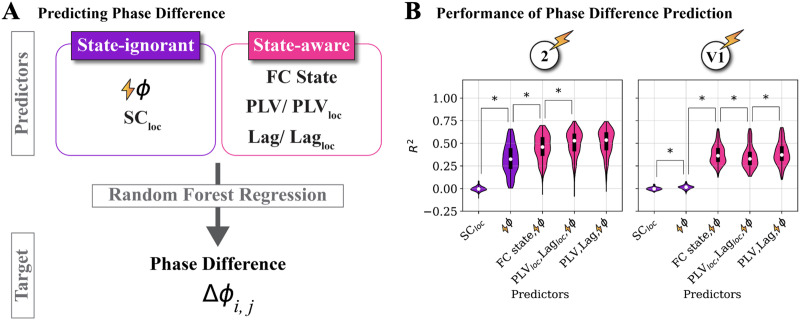
Stimulation effects are better extrapolated by FC state-aware prediction. Different structural and dynamical features have different impact on the effects of external stimulation. To determine their relative importance we train random forest regression (RFR) machine-learning algorithms to predict phase-shifting effects. (A) Top: Predictors for the regression can contain: “FC-state ignorant” features (purple) such as stimulation phase and outgoing structural connectivity of the stimulated region (SC*_loc_*); or “FC-state aware” features (pink) such as the discrete label of the inferred FC state, the PLV, and lag links with regions that the stimulated region was structurally projecting to (PLV*_loc_*, Lag*_loc_*), or the full PLV and lag matrix. Bottom: These predictors can be flexibly concatenated to predict the stimulation induced phase shifting in the desired region *j* when stimulating region *i*. (B) Prediction performance of stimulation-induced phase shifting. Violin plots reflect the test performance of 10 cross-validation folds for 29 affected regions (*n* = 290 points in total; white dots mark the medians, thick black line the interquartile range). Including state-aware predicting features significantly improved the prediction (Mann–Whitney *U* test, * = *p* < 0.05, Bonferroni corrected). Significance of comparisons are reported only for adjacent distributions (Bonferroni correction does nevertheless account for all possible comparisons, detailed in Supporting Information Figure S9A).

We compare the prediction capabilities of input variables that are “state-ignorant” versus “state-aware” (see purple and pink boxes in Figure 6A). The former describes local features like the phase of the stimulated region (with reference to its own oscillation) at the time when stimulation is applied. We also consider the outgoing structural connectivity of the stimulated regions. The rest of the variables describe the global FC state at the time of stimulation in various levels of detail like the FC state identity, PLV, and lag matrices (or summary metrics derived from them).

The predictive power is significantly improved by the state-aware variables (pink) with respect to the state-ignorant variables (purple), as shown by the comparison of prediction performances in Figure 6B (for stimulation in two example regions, V1 and 2, see Supporting Information S9A for other regions). For the state-ignorant variables, stimulation phase outperformed the outgoing structural connectivity of the stimulation region local structural connectivity SC_loc_ for all stimulated regions (except DP). Furthermore, when including state-aware features, performance increases up to ~35% for stimulating region 2 and region V1. Note that the relative performance of state-ignorant and state-aware predictors heavily depends on the stimulated region, with strongest improvements for V1, 2, 8B, and STPr (~20% to ~40%), compared to moderate ones for regions DB and TEO, in a range between ~7% to ~14% (Supporting Information Figure S9A). Specifically, we considered three possible alternative state-aware inputs. First, we used just the discrete label of the FC state (Ψ_1_, … Ψ_3_) attributed to the time epoch in which stimulation is applied. Even such a rough indication of the whole network dynamics is already enough to significantly improve the performance as compared to state-ignorant prediction. A further improvement is obtained by using: second, the description of the local neighborhood of the stimulated node in the FC network, characterized by the PLV values and the phase lags of this node i with all the other regions structurally connected to it (i.e., the i-th rows of the time-epoch-specific PLV and lag matrixes); or, third, the entire time-epoch-specific PLV and lag matrices (including as well PLV values and lags between pairs of regions both remote from the stimulated node). Interestingly, the size of improvement from state-ignorant input variables (stimulation phase) to extracted FC states labels and PLV and lag matrices was not systematic across stimulated regions. For example, there was no significant improvement with full PLV and lag matrices with respect to the discrete FC states in V1, whereas region 2 improved with every more fine-grained state-aware input variable. This suggests that for some regions, limited information is lost by discretizing the dynamical repertoire into FC states. Another point is that adding only the structurally connected PLV and lag values as state-aware predictors generally performed as well as using the whole PLV and lag matrices (with exception of V1). The two choices of input features may end up not being very different, given how dense the SC matrix is (cf. Figure 2A). However, another possibility is that, indeed, accounting for the interactions of the stimulated node with just its local neighbors provides a reduced description sufficient to capture system’s level complexities. In the context of statistical physics, a description in which the local neighborhood of the stimulated node is parameterized corresponds to a so-called “Bethe-Peierls” approximation (Bethe, 1935). This level of description is already more detailed than a “mean-field” approximation in which the global network state is just compressed out into a single label (i.e., FC state identity). It is however less detailed than the “exact” description in which the system’s state is represented in full detail by the complete PLV and lag matrices. Thus, we could summarize by saying that for predicting the effect of stimulation: a mean-field account of the system’s state is already better than ignoring it; and that a Bethe-Peierls account of the system’s state may already be sufficient for further improvement without the need to go toward an exact description (see Discussion).

Lastly, the results shown in Figure 6B report prediction performances irrespective of the state in which the system was when stimulated. It is possible, however, that the classifiers perform better when stimulating the system in certain states rather than others. In other words, the predictability of stimulation effects may be higher or lower depending on the system’s state. We thus evaluate prediction performances for each of the stimulated FC states separately. As shown in Supporting Information Figure S9B, for all stimulation states, differences in performance are not systematic. Therefore, we conclude that the superior performance of state-aware predictors with respect to state-ignorant predictors holds in general and does not depend on state-ignorant classifiers performing poorly in some of the states.

## DISCUSSION

This paper analyzes the contributions of global and local dynamics on the long-term effects of single pulse stimulation in a large-scale computational brain network model. To do so, we characterize the complex oscillatory behavior of the resting state by detecting transient network activity states in the dynamic repertoire of the model based on phase-locking patterns between all regions. Thereafter, we show that stimulation effects depend on the state of the network at the stimulation time. Stimulation induces either state morphing, meaning phase modifications while remaining in the same network state, or state switching, thus deviating from the resting-state trajectory to reach a different state. In both cases, stimulation effects jointly depend on stimulation phase, network state, and region. Finally, we demonstrate that including state-aware information during the prediction of stimulation-induced phase shifting improved performance in predicting stimulation phase shifting effects, validating the importance of this often-neglected global information in the understanding of stimulation effects.

Our results are in line with previous experimental studies that have shown, independently, that stimulation phase (Duchet et al., 2020; Momi et al., 2022; Momi et al., 2023; Peles et al., 2020; Poorganji et al., 2021; Zrenner et al., 2018) and global oscillatory states (Janssens & Sack, 2021), modulated by cognitive or emotional tasks (Croce, Zappasodi, Spadone, et al., 2018; Isserles et al., 2011), can affect the efficacy of stimulation. Furthermore, our complex oscillatory model shows that these factors exhibit intricate interdependencies and should thus be jointly considered when evaluating the stimulation responses in the brain.

The nonlinearity in our system limits the applicability of linear control theory as it transcends temporal dynamics such as exponential growth, exponential decay, and sinusoidal oscillations, which can be accounted for in the framework of network control theory (Srivastava et al., 2020). Additionally, tools for inferring causality are strongly grounded in linear systems theory and can only be applied if a system behaves linearly (Wagner, 1999). Nevertheless, we can apply the notion of causality in subspaces of nonlinear systems that behave in an approximately linear manner (Mitchell, 2008). Here, we provide a first characterization of complex dynamics in terms of FC states, with internally (relatively better) preserved inter-regional phase relations. In this way, we can see FC states as different local “patches” within the broader high-dimensionality space in which the system’s nonlinear dynamical flow unfolds (Figure 3C, left) into which a prediction of the stimulation effects can still be performed in terms of the FC state (i.e., patch)-specific effective PRC. In this way, we can still model causal interdependencies state by state, which improves prediction significantly for all regions, with respect to state-ignorant attempts to anticipate stimulation effects (Figure 6B).

Note that our definition of “state” is specific, linked to tracking the transient dynamics of regional activity via FC (Hutchison et al., 2013). This is in contrast with alternative notions in the literature. In experimental studies, the term “state” has been used more often to refer to states of consciousness such as wakefulness, sleep, or anesthesia (Blume et al., 2015; Bradley et al., 2022) or behavioral states such as resting or task state (Gonzalez-Castillo et al., 2015; Xie et al., 2018). The dependency of stimulation effects on dynamical states had also been addressed by previous computational works (e.g., Papadopoulos et al., 2020); however, these previous works primarily explored alternative choices in global dynamical WP, closer or farther from some critical point and did not focus on the temporal dynamics. Here, our network activity states correspond to transient configurations of FC, which are sampled by resting-state dynamics over the slow time scales of fMRI dFC (tens of seconds; Allen et al., 2014; Battaglia et al., 2020; Cabral et al., 2017) or the faster EEG and LFP (Local Field Potentials) microstate switching (several hundreds of milliseconds to a few seconds; Clawson et al., 2019; Pedreschi et al., 2020; Van de Ville et al., 2010). Stimulation-induced state morphing and state switching effects are achieved on scales of one or more oscillation cycles, that is, on times that are short with respect to most spontaneous state transitions. Therefore, future studies could be designed to allow explicit identification of states and state dependencies of stimulation effects, investigating a previously neglected source of variability.

Our results may also call for a reinterpretation of the mechanisms behind the stronger efficacy of repeated stimulation. Stimulation studies are preferably conducted with repetitive stimulation as the effect is larger and more persistent, a fact often attributed to plasticity changes (Iaccarino et al., 2016; Schwab et al., 2021). However, our model without plasticity hints at an alternative mechanism that could explain the superior efficacy of repeated stimulation, without the need of invoking plasticity. Indeed, repeated stimulation could induce a cascade of state switching that would lead the network toward the most temporally stable states (reinforcing attractors in a sense, as is the case for chains of stochastic transitions). It could also “pin” the system in a given state, similar to entraining phase oscillators (Strogatz et al., 1989). This enhanced stability occurs on a fast timescale, but could of course interact with plasticity (at various timescales) to bring further dynamic self-organization.

Remarkably, our simulations and theory also suggest that long-lasting effects could also be achieved with single pulse stimulation, provided that the timing of this stimulation is properly chosen, accounting for ongoing local and global dynamics. Our model indeed predicts that the effect of single pulse stimulation will be weak for most combinations. To overcome the “rigidity” of the brain and failure to elicit effects (López-Alonso et al., 2014), one needs to identify specific “dynamic hotspots” in a sea of apparent randomness. Detecting such hotspots in practice calls for an online tracking of dynamic FC state prior to stimulation (Kraus et al., 2016; Ngo et al., 2013; Rosin et al., 2011; Witt et al., 2013), which may be feasible if states dictionaries are pre-extracted from resting-state time series and simple distinctive features identified (e.g., state-specific hubness of a few reference regions, cf. Figures 3A and 3D).

In our study, we made the operational assumption that discrete FC states can be defined (Figure 3), an assumption that led us to the distinction between state morphing and state switching, following terminology first introduced by Papadopoulos (2021). However, whether FC states are discrete or continuous is a long-standing debate in the dynamic FC literature. Many authors have used unsupervised clustering to extract discrete FC states (e.g., Allen et al., 2014; Thompson & Fransson, 2016) and alternative formalisms describing dynamic FC as a smooth reconfiguration process have been proposed (e.g., Battaglia et al., 2020). Here, we show that characterizing FC states as discrete states is already enough to improve the prediction of stimulation-induced effects (Figure 6B). However, characterizing FC states as continuous by using raw instantaneous measures such as PLV and lag (instead of discrete FC state labels) can further improve prediction, at least for some regions. From a dynamical systems perspective, this graded variation between discrete-like and continuous aspects in FC dynamics is also compatible with expectations from the Structured Flows on Manifolds theory (Jirsa, 2020) or effective free energy landscape models (Ezaki et al., 2017). Discrete states that allow for separating state morphing from state switching can be seen as an extreme (i.e., thresholded) version of the characterization of short versus long excursions (with a metric to be defined). As an example, the variability in state persistence across time is reminiscent of the alternation between metastable “knots” and fast reconfiguration “leaps” previously described in resting-state dynamic FC (Battaglia et al., 2020).

In network models with more complex dynamics, intra- and interstate variability may not only be reflected in different phase configurations, but also in frequency and amplitude profiles as well. Frequency may be particularly important for the case of rhythmic stimulation, as it can induce frequency-specific state changes (Leuchter et al., 2021; Vosskuhl et al., 2018) and cross-frequency effects beyond the targeted frequency band (Ozdemir et al., 2020), as predicted by other models (Papadopoulos et al., 2020). In our analysis, we do not consider frequency variability and cross-frequency effects. However, our model is not a simple network of Kuramoto-like phase oscillators with prescribed frequency (as in, e.g., Cabral et al., 2011). On the contrary, oscillations are emergent, and their frequency is dynamically tuned by the received inputs. As such, our model does, in principle, give rise to complex frequency modulations that are intrinsically coupled as they originate from the collective dynamics of the same nonlinear dynamic system. Future studies could expand our analysis for characterizing dynamic cross-frequency coupling, that can be rigorously studied in exactly reduced models (Ceni et al., 2020) similar to the ones we use by using analytically estimated population-level PRCs (Dumont et al., 2017).

Prestimulus amplitude is another important aspect that may be considered in stimulation in conjunction with phase, according to recent experimental studies: beside phase, stimulation effects can depend exclusively on oscillatory power (Ferreri et al., 2014; Iscan et al., 2016; Schulz et al., 2014), the interaction of phase and power (Hussain et al., 2019; Khademi et al., 2019) or neither of them (Madsen et al., 2019) exhibiting an ambiguity reminiscent of the complexity displayed in our study. Again, this could be studied in our model in the future as it displays intrinsically coupled phase and amplitude fluctuations, unlike simpler networks of phase oscillators.

Following this line of thought, it is worth noting that extensions of the PRC concept that can account for amplitude variations have already been extensively studied (Castejón et al., 2013). Indeed, using mathematical tools that extend the conventional assumptions behind standard PRCs (isolated node, phase-reduced node dynamics), descriptions of the stimulus response of an oscillating population embedded within a larger network could be potentially derived in a mathematically rigorous manner. As shown by Dumont and Gutkin (2019), it is possible to rigorously define a “global PRC” that quantifies how the phase-shifting effect induced by stimulation propagates from one population to other populations, in a network of phase-locked oscillating populations. Such analytical treatments have only been proposed for toy models made up of only a few regions, in line with previous work that showed how PRC depends on intra-regional E and I coupling strengths or synaptic delays (Battaglia et al., 2007; Dumont et al., 2017; Dumont & Gutkin, 2019). However, this approach may quickly become infeasible as soon as the number of coupled populations increases and the coupling between them becomes heterogeneous and irregular, as in typical connectome-based models. Here, we deliberately chose to abandon formal rigor to introduce an operational notion of effective PRC instead. They are not analytically evaluated from the equations of network dynamics, but empirically measured in simulated stimulation experiments. The advantage of this pragmatic approach is that effective PRCs can be used to describe the phase dependency of stimulation effects to an embedded node “as if” this node was isolated. The drawback is that these effective PRCs become inherently state dependent; in other words, we reduce the complexity of collective dynamics into a (small) number of states.

We chose a “well behaved” dynamic WP to increase the probability of falsifying our central hypothesis and finding that a description in terms of just one classic PRC is still valid, because of the high overall synchrony in the network. This means that stable inter-regional phase locking patterns may lead to robust phase-dependence in the effects of stimulation. Ultimately, however, even in such a well-behaved WP, we encountered a tremendous amount of complexity, which justified the need of multiple effective PRCs. We did not perform simulations in other WPs, because the numerical experiments were computationally heavy. Regardless, we expect less synchronized regimes to involve further complexity, making our results even more pertinent. Another ingredient missing in our model are delays (Petkoski & Jirsa, 2019), which would also increase complexity as they favor symmetry-breaking, out-of-phase locking, and multistability leading to a larger number of states in the dynamic repertoire (Battaglia et al., 2012; Dumont & Gutkin, 2019). Future studies could also investigate scenarios in which stimulation, beyond altering transient exploration within the system’s dynamic working point, modifies the working point itself—an even more extreme form of state switching in which the entire landscape of available states becomes reconfigured. Such “dynamic landscape morphing” could be monitored through changes in overall metastability and in the complexity of state transitions (Bapat et al., 2024).

Overall, our model remains very abstract with respect to several details. Firstly, we are modeling stimulation as a positive, instantaneous, and perfectly localized pulse in an attempt to emulate Dirac’s delta in the classic PRC. In practice, stimulation has a temporally heterogeneous shape and may be positive and negative over the time course of the stimulation (Vosskuhl et al., 2018). Furthermore, the primary area of stimulation may not be spatially localized, but rather spread out (Polanía et al., 2018). These aspects may be taken into account by future studies and easily modeled, for example, using already implemented features of The Virtual Brain (TVB) neuroinformatic platform (Sanz Leon et al., 2013), such as its built-in stimulus editor and surface-based neural field simulator. Another limitation is the strong intensity of the stimulation pulse relative to the ongoing resting-state oscillation. However, we expect that weaker more realistic stimulation intensities could still be effective when applied in the “dynamic hotspots,” because the stimulation effect (Figure 1C, red and blue dashed line) may be amplified by intrinsic self-organized dynamics (Figure 1C, red and blue solid line). Secondly, oscillations produced by our model are highly regular, enabling phase estimation through simple linear interpolation methods—an advantage also for future closed-loop implementations (Witt et al., 2013). For empirical data, however, a Hilbert-transform–based extraction of protophases, followed by correction toward true phases with homogeneous rotation speed (Kralemann et al., 2008), would likely constitute a safer and more robust strategy. Thirdly, we use a very dense and nonhuman connectome. However, similar dynamic complexity has been replicated with human connectomes of a lower density (Hansen et al., 2015; Rabuffo et al., 2021).

In conclusion, this study could serve as a first step toward using computational models and dynamic aware prediction algorithms to improve stimulation protocols. As an example, single-trial TMS variability is traditionally discarded as noise and averaged out. Our study points toward the possibility that this variability could be a signature of complex dynamics and a useful target for optimizing the efficacy of stimulation. In the future, spontaneous and stimulation-induced variability could be used as a fitting target for the construction of personalized models of the stimulation effects in specific patients (as already attempted in the modeling of epileptic seizure spreading, cf. Proix et al., 2017), with improved parameter identifiability (Domhof et al., 2022; Wischnewski et al., 2022). Such personalized models of nonlinear brain dynamics could be adopted to design tailored protocols of stimulation by targeting the patient-specific dynamic hotspots and increasing energy efficiency by reducing the number and intensity of the applied pulses. We expect personalized virtual brain models to achieve better performances in designing control schemes for brain states, beyond current proposals based on linear control theory (Muldoon et al., 2016). Last but not least, the personalized fitting of virtual brain models to spontaneous and stimulated activity could enable us to reverse engineer directly linked physiological parameters of interest. Indeed, in exactly reduced models as the ones we use here to describe regional dynamics, parameters in the macroscopic population activity-level description can be precisely related to microscopic parameters as neural excitability and synaptic conductance (Montbrió et al., 2015). As stimulation can also be used for revealing potential pathologies (Casali et al., 2013), we can link the altered stimulation responses to maladaptive circuit mechanisms inferred via model fitting.

## MATERIALS AND METHODS

We implemented all the simulations in TVB (https://www.thevirtualbrain.org) software, a framework for the simulation of dynamics in large-scale brain networks, constrained by an imposed structural connectome, with customizable choice of local regional dynamics and possibility to apply arbitrary spatiotemporal patterns of stimulation (Sanz Leon et al., 2013).

### Structural Connectivity

Due to computational constraints, we selected a small, but dense structural connectivity matrix (29 areas; see Figure 2A). The anatomical connectivity was obtained from 29 macaque monkeys through retrograde tracing and is weighted as well as directed (Markov et al., 2014).

### Neural Mass Model

To model a single brain region, we coupled an I and E population described by a mean field approach obtained from an exact reduction as described in (Dumont & Gutkin, 2019). This model is implemented in TVB under the regional dynamics of the built-in type DumontGutkin. Within each region, an E and an I population were coupled similar to a PING configuration with a local drive Iext to the E population (see Figure 2B), which is a canonical description of cortical oscillations in the low gamma frequency range (Dumont & Gutkin, 2019). Unlike the traditional PING, our configuration also features a local drive *I*_*ext*_ to the inhibitory population. However, the local dynamics of this PING-inspired configuration remained similar to the local dynamics of the traditional PING configuration as revealed by stimulation of this configuration in isolation.

The excitatory firing rate of each neural mass was coupled with the E and I populations of all other structurally connected neural masses. Stimulation entered the neural mass as an additive term in the average membrane potential *V_e/i_* similar to the local drive *I*_*ext*_. Such average membrane potentials, time series of which are shown in Supporting Information Figure S1, can be interpreted as sources of an externally measured EEG signal, following a common interpretation prevalent in the modeling literature (Jirsa et al., 2002; Ritter et al., 2013). Individual inter-regional connections strengths were dictated by the structural connectivity matrix scaled by adjustable free parameters. Long-range inter-regional excitatory connections targeted both the E and the I postsynaptic populations, with distinct scaling factors *G*_*ee*_ (for excitatory-to-excitatory coupling) and *G*_*ei*_ (for excitatory-to-inhibitory coupling). The latter is unusual for computational models implemented with the TVB software and thus required a specific adaptation of the source code. Such excitatory-to-inhibitory coupling, besides its realism, contributes to make the collective dynamics of the model richer and more complex. In reference to well-documented ratios of E to I populations in the brain, the input to the inhibitory population is scaled to be five times larger than *G*_*ee*_ (Sahara et al., 2012). All connections were instantaneous (no delay was used) and all simulations deterministic (no noise). Therefore, all FC variability stems from dynamic complexity (quasi-periodicity, chaos…) rather than from stochasticity of inputs.

$$\left\{\begin{array}{c} \begin{array}{c} \frac{d}{dt}r_{j}^{e}=\frac{1}{\tau_{e}}\left(\frac{{∆}_{e}}{\pi \tau_{e}}+2V_{j}^{e}r_{j}^{e}\right)\\ \frac{d}{dt}V_{j}^{e}=\frac{1}{\tau_{e}}\left({V_{j}^{e}}^{2}+\eta_{e}+I_{\mathrm{ext}}+I_{\text{stim}}+\tau_{e}s_{ee}-\tau_{e}s_{ei}-\tau_{e}^{2}\pi^{2}{r_{j}^{e}}^{2}\right) \end{array}\\ \begin{array}{c} \frac{d}{dt}s_{j}^{ee}=\frac{1}{\tau_{s}}\left(-s_{j}^{ee}+J_{ee}r_{j}^{e}+G_{ee}\sum_{j\neq k}{SC}_{jk}r_{k}^{e}\left(t-1\right)\right)\\ \frac{d}{dt}s_{j}^{ei}=\frac{1}{\tau_{s}}\left(-s_{j}^{ei}+J_{ei}r_{j}^{i}\right) \end{array} \end{array}\right.$$

And

$$\left\{\begin{array}{c} \begin{array}{c} \frac{d}{dt}r_{j}^{i}=\frac{1}{\tau_{i}}\left(\frac{{∆}_{i}}{\pi \tau_{i}}+2V_{j}^{i}r_{j}^{i}\right)\\ \frac{d}{dt}V_{j}^{i}=\frac{1}{\tau_{e}}\left({V_{j}^{i}}^{2}+\eta_{i}+I_{\mathrm{ext}}+I_{\text{stim}}+\tau_{i}s_{ie}-\tau_{i}s_{ii}-\tau_{i}^{2}\pi^{2}{r_{j}^{i}}^{2}\right) \end{array}\\ \begin{array}{c} \frac{d}{dt}s_{j}^{ie}=\frac{1}{\tau_{s}}\left(-s_{j}^{ie}+J_{ie}r_{j}^{e}+\Gamma *G_{ee}\sum_{j\neq k}{SC}_{jk}r_{k}^{e}\left(t-1\right)\right)\\ \frac{d}{dt}s_{j}^{ii}=\frac{1}{\tau_{s}}\left(-s_{j}^{ii}+J_{ii}r_{j}^{i}\right) \end{array} \end{array}\right.$$

With four state variables per population for region *j*: the firing rate *r*_*e*_ of the E and *r*_*i*_ of the *I* population, the average membrane potential *V*_*e*_ of the E and *V*_*i*_ of the I population, the synaptic currents projecting from E-to-E population (*s*_*ee*_), E-to-I population (*s*_*ie*_), I-to-I population (*s_ii_)*, and I-to-E population (*s*_*ei*_). Other parameters are the spread of the heterogeneous noise distribution of the E population Δ_*e*_ = 1 and the I population Δ_*i*_ = 1, the mean heterogeneous current to the E population *η_e_* = −5 and to the I population *η_i_* = −5, the characteristic time constant of the E population *τ_e_* = 10 and the I population *τ_i_* = 10, the synaptic time constant *τ_s_* = 1, the synaptic weight between the I population and the E population *J*_*ei*_ = 19 and between the E and I population *J*_*ie*_ = 19, the ratio *G*_*ei*_ and the inhibitory global coupling to *G*_*ee*_ the excitatory global coupling Γ = 5, and the structural connectivity SC as described in the previous section. Finally, the two parameters we explore in search of a WP: the external homogeneous current to the E population *I*_*ext*_ and the excitatory global coupling *G_ee_.* The equations were adapted from Dumont and Gutkin (2019) and can be consulted for further details how this neural mass was obtained through an exact reduction from spiking models.

### Parameter Exploration: Dynamical Measures

To identify the different regimes of collective dynamics produced by our model, we performed simulations of spontaneous (unstimulated) dynamics systematically varying two free parameters over a prescribed range of values. We explored the global excitatory-to-excitatory coupling *G*_*ee*_ versus the local excitatory drive *I*_*ext*_ and chose a dynamical regime (*G*_*ee*_ = 80.0; *I*_*ext*_ = 6.0) at the cusp between clearly defined regimes identified through inspection of various metrics of the generated dynamics of the firing rate in the E population: rate of activity, and its variability across time and between regions; and PLV (Figure 2C). We now describe in detail these metrics used to track alternative dynamical regimes of the model.

#### Rate.

To quantify the overall level of activity in the network, we calculated *μ_j_*, the average rate of activity across time for each region. We then took the median across regions of these regional time averages (see Figure 2C, top left):

$$\mu_{j}=\frac{\sum_{t=1}^{T}r_{jt}}{T}$$

where *r*_*jt*_ corresponds to the excitatory rate of region *j* at timestep *t* and *T* corresponds to the total number of timesteps in the time series. We then define the score *R*_*tot*_ as the median over regions of the values *μ_j_*. Larger values of *R*_*tot*_ indicate larger overall levels of activity within the network.

#### Rate heterogeneity across regions.

To assess whether all regions have similar or different levels of activity, we calculate the coefficient of variation (CV) of the region-specific time-averaged rates *μ_j_* (see Figure 2C, bottom left), that is, the ratio between the standard deviation and *μ_tot_* the mean across regions of the time-averaged rates of each region:

$$\mu_{tot}=\frac{\sum_{j=1}^{J}\mu_{j}}{J}$$

$$R_{het}=\frac{\sqrt{\frac{\sum_{j=1}^{J}{\left(\mu_{j}-\mu_{tot}\right)}^{2}}{J}}}{\mu_{tot}}$$

where *μ_j_* corresponds to the average rate of activity across time for each region *j, J* corresponds to the total number of regions, and *μ_tot_* corresponds to the grand-average rate. Larger values of *R*_*het*_ indicate larger heterogeneity of average rate across regions.

#### Rate variability across time.

To capture the overall amplitude of oscillations in the network activity, we calculated, for each region, the CVs of rate along time, that is, the ratio between the standard deviation and the average over time of the regional activity rate. We then took the median across regions of these CVs across regions (see Figure 2C):

$${cv}_{j}=\frac{\sqrt{\frac{\sum_{t=1}^{T}{\left(r_{jt}-\mu_{j}\right)}^{2}}{T}}}{\mu_{j}}$$

where *r*_*jt*_ corresponds to the excitatory rate of region *j* at timestep *t, T* corresponds to the total number of timesteps in the timeseries, and *μ_j_* corresponds to the average rate of activity across time for each region *j*. We then define the score *R*_*var*_ as the median over regions of the values *cv*_*j*_. Larger values of *R*_*var*_ indicate oscillations with larger amplitude of relative variation between oscillation minima and maxima.

#### PLV.

To capture the overall level of phase synchronization depending on the dynamic regime, we calculated the PLV among all pairs of regions (PLV_all_), which can disentangle phase co-fluctuation from amplitude co-fluctuation (Lachaux et al., 1999). The phase of an oscillation was obtained by performing a linear interpolation between the troughs of the oscillation to remove any effect of amplitude modulation. The troughs were detected by inverting the oscillation and using the find_peaks function from the scipy library (Version 1.3.1). After extracting phases, we then evaluated the PLV for each pair of regions as:

$${PLV}_{j,k}=\frac{1}{T}\left|\sum_{t=1}^{T}e^{i\left(\phi_{jt}-\phi_{kt}\right)}\right|$$

where *ϕ_j/kt_* corresponds to the instantaneous phase of region *j/k* at timestep *t* and *T* corresponds to the total number of timesteps in the time series. PLV values can range from 0 (no phase locking) to 1 (perfect phase locking) and is undirected as well as symmetric.

$${PLV}_{all}=\frac{\sum_{k}^{J}\sum_{j}^{J}{PLV}_{jk}}{\frac{J\left(J-1\right)}{2}}\text{for k}>j$$

Where *PLV*_*jk*_ corresponds to the PLV between region *j* and *k* and *J* corresponds to the total number of regions. Due to the symmetric nature of PLV, we only calculate PLV_all_ based on each unique PLV pair (*PLV*_*12*_ = *PLV*_*21*_). An indication of the global level of synchrony over the entire network is obtained by averaging PLVs over all pairs of regions, yielding the PLV_all_ measure. It is also possible, however, that strong phase synchrony exists between a subset of regions, despite global synchrony being overall low. To capture such a potential scenario, we also evaluated the quantity PLV_top_, which is the average of pairwise PLVs over the top quartile of pairwise links with the highest PLV values.

### Simulating FC Dynamics and Extracting FC States

#### Resting-state simulation.

We used a deterministic modified Euler’s method (Heun method with zero noise setting in the TVB software option choices for integrator) with a very precise integration timestep (0.00005 in TVB settings) to maintain stable integration across the application of very fast stimulation. The generated time series were then down sampled, saving only one point every 200 integration steps (i.e., every 0.01 timesteps). We cut off an initial transient of 400 steps and simulated a time series of 3,000 timesteps for 13 randomly generated initial conditions. To calculate dynamic FC measures, we used the sliding window method to obtain 140 steps windows with a 75% overlap, resulting in 1,066 windows across all resting-state simulations. We chose this window length in order to capture at least 50 oscillation cycles within each window, at the retained dynamic WP.

#### Time-resolved characterization of FC.

In every window, we computed the value of PLV (strength of phase locking) for each pair of regions and the phase difference at which their phase locking occurred (lag of phase locking, see later). We then compiled these PLV and phase lag values into two matrices that, together, provide a characterization of the instantaneous FC configuration.

#### Hubness and hubs.

To distinguish regions that tended to participate in phase-locked coalitions, that is, being strongly phase locked with other regions in our simulations, we calculated a measure of “hubness” for each region (Supporting Information S2) based on time-resolved FC analyses. First, we binarized the weighted graphs described by each time resolved PLV matrices, retaining only links with PLV strength above a certain threshold. This choice naturally separated two ensembles of links naturally emerging from our simulations. Indeed, as shown by Supporting Information Figure S2A, the distribution of median PLV for different pairwise links has two clear peaks, one at a low PLV value of ~0.2 and a second with a high PLV value close to ~0.9. Both links at high and low median PLV can display a variability of the phase lag at which phase locking occurs. Inspection of the median-versus-variance PLV scatter plot in Supporting Information Figure S2B reveals a clear separation between two clusters: one consisting of links with relatively higher median PLV and substantial variability, and another comprising low-median, high-variability links. The latter likely reflects variability driven primarily by noise, whereas the former contains the links that meaningfully contribute to the structure of dFC. We therefore select a PLV-median threshold based on the distribution in Supporting Information Figure S2B to filter out noise. Specifically, retaining only links above the 54th percentile, PLV threshold removes weakly locked links—whose phase lags are noisy and ill defined—while preserving strongly locked links that still exhibit rich variability in their phase relations.

Then, we calculate hubness, defined as the degree of a node (i.e., number of strong links) in each given binarized PLV window (Supporting Information S2C). Hubness thus provides an integer-valued metric of time-resolved centrality for a node. The most degree-central nodes in every timeframe were denoted as “hubs.” To discriminate hub from nonhub regions in each given window, we applied a second thresholding, this time not based on the PLV strength of the links but on the degree of the nodes in the binarized PLV network frames. Specifically, regions were denoted as hubs if their hubness was equal to or above 21. This hubness threshold guarantees that regions that have at least one hub also have a large average PLV value (green line in Supporting Information Figure S2D) and, at the same time, a sufficient variability of hubs across time on average (orange line in Supporting Information Figure S2D). This allows for the variability of hub assignments across windows, which is necessary for clustering, while for lower (higher) thresholds, variability would be nonexistent, because all (no) regions are labeled as hubs across time.

#### Phase lag.

To calculate the lag of phase locking between pairs of regions in each window, we take the firing rate of the E populations in each region and calculate their cross-correlogram, describing how the Pearson correlation between the time series varies as a function of variable latency shift. Phase lag, normalized in the [0,1] interval (with 0 denoting in phase and 0.5 anti-phase phase locking) is determined as the ratio between the latency of the first off-zero peak and the interval between the first and the second peaks (giving the average oscillation period). Cross-correlograms were smoothed (via low-pass filtering in Fourier space) to simplify peak detection. To reduce the noise in the lag measure, we only compute phase lags between stable hub regions, that is, regions labeled as hubs at least once in every hub cluster (see FC states: characterization section), that is, overall, 66 pairwise links considered for lag analyses.

#### FC states: Characterization.

After characterizing the FC in every time window in terms of its hubness and phase lag profiles, we grouped time windows into discrete states using unsupervised clustering, in line with a variety of methods for dFC characterization (Hutchison et al., 2013). We performed two parallel clustering, one in terms of instantaneous hubs (i.e., the 29-dimensional binary vectors of which regions are labeled as hub or not in the considered window) and a second in terms of phase lag profiles (i.e., the 66-dimensional vectors of phase-lags across all pairs of stable hub regions). We used the AgglomerativeClustering hierarchical clustering algorithm from the Scikit learn library Version 0.21.3 (Pedregosa et al., 2011), which starts by treating each object as a singleton cluster and then successively merges pairs of clusters based on their similarity forming a tree-based representation of their distance inter-relations. The procedure continues until all clusters have been merged into one big cluster containing all objects. The resulting dendrogram is then thresholded to yield the desired number of clusters. Based on an average silhouette criterion (Rousseeuw, 1987), we chose a number of five clusters in terms of hub vectors (Figure 3A, right). Since FC states must have characteristic hub and lag profiles simultaneously, we chose four clusters in terms of phase lag vectors (Figure 3B, right). Then, we constructed a contingency table between the two alternative hub- and lag-based clusterings (Figure 3C, right). Hub cluster “4” with lag cluster “1”, hub cluster “3” with lag cluster “4” and hub cluster “2” with lag cluster “2” gave rise to the three largest intersecting subsets. We thus took the time windows belonging to these three intersection subsets and assigned to them, respectively, the FC state labels Ψ_1_, Ψ_2_, and Ψ_3_. All windows that did not fall inside these overlapping intersections were indistinctly termed “None.” Such construction of FC states, indeed, does not aim at attributing a state label to every possible window, but, on the contrary, at identifying sets of highly specific windows whose FC hubs and lags profiles are maximally consistent within groups and maximally distinct between groups.

To visualize similarity and differences across the FCs in different time windows, we concatenated hubs and phase lag vectors and applied on these concatenated vectors a t-SNE (van der Maaten & Hinton, 2008) unsupervised algorithm for dimensionality reduction. The resulting nonlinear embedding provides a two-dimensional scatter plot in which the FC of every window is mapped to a point and in which smaller or larger distances across projected points in the 2D plane relate to higher or lower similarities across FCs in the original (29 + 66)-dimensional space (Figure 3C, left).

#### FC states: classification.

Even after applying the careful selection previously described, there is residual variability across the time windows within each of the states Ψ_1_, Ψ_2_, and Ψ_3_. Furthermore, when applying stimulation, the system generates previously unseen FCs for which there is no state assignment available from unsupervised clustering. It is thus necessary to identify FC windows maximally representative of the specific state to which they belong (state “prototypes”) and to construct a supervised classifier that is able to assign a state label to unseen windows based on their degrees of similarity to the different state prototypes. To quantify how representative of an FC state each time window is, we summed the two silhouette scores (Rousseeuw, 1987) of the window obtained independently for hub-based and lag-based clusters. The silhouette score values range between 1 (best) and −1 (worst) and quantify how similar a sample is to its own cluster relative to how similar it is to other clusters. For each state, we thus select 100 windows with the highest *typicality* (additive silhouette score) per state.

To identify the FC states in novel poststimulation samples, we constructed a *k-*nearest neighbors classifier using these 100 prototypes per state as training set. This classifier identifies the *k* nearest training set samples for a novel sample and assigns the novel sample the state label to which the majority of its nearest neighbors belong to. A choice of *k* = 50 guaranteed the best generalization performance over the remaining nonprototypical resting-state windows for which a ground truth state label was available.

### Stimulation

#### Stimulation simulation.

We applied a punctual single pulse stimulation to emulate Dirac’s delta applied to both the I and E populations of the stimulated region and simulated 500 timesteps poststimulation. Stimulation was systematically applied at different phases of the ongoing oscillation cycle, from 0 to 2*π* in 10 intermediate steps. We used a stimulation intensity of 300 (2 magnitudes larger than the adopted background drive *I*_*ext*_) and performed stimulation experiments in six different regions (V1, DP, TEO, 2, 8B, STPr), and three different FC states (Ψ_1_, Ψ_2_, Ψ_3_). Such stimulation intensity was empirically selected from five tested values (75, 150, 300, 450, 600 in our arbitrary units) because it satisfied two criteria: it was the smallest intensity that elicited a range of phases with significantly increased state-switching probability when stimulating each of the targeted regions (cf. Figure 5D, Supporting Information Figure S8); and it was the largest intensity for which there still existed phase bins without significant phase shifting in all monitored regions for all stimulated sites (cf. Figure 5C, Supporting Information Figure S7). In other words, we chose this value because it was neither too weak nor too strong (see Supporting Information Figure S4 for comparison with PRCs obtained at other stimulation intensities). For each FC state, we ran simulated stimulation experiments in five different time windows (the five prototype window with the topmost values of combined silhouette, see previous section) and in five subsequent oscillation cycles, resulting in 25 simulations samples in total for each combination of region, phase and state, provided that they lay 500 simulation steps before the end of the resting-state simulation. Due to computational tractability, we had to make a subselection of regions to stimulate in our simulation. The rationale for choosing the six regions we considered was the following: We covered most cortical areas as TEO is in the visual area, STPr is in the motor area, DP is in the parietal area, and 8B is in the prefrontal area; furthermore, to investigate regions with two extreme roles with respect to FC topology, we also stimulated V1, which has the lowest probability serving as hub and 2, which has the highest probability.

#### Phase shifting portraits.

We obtained the phase-shifting portrait of each stimulation simulation by calculating the phase difference for each stimulated phase between stimulated and unstimulated time series at each of the 500 timesteps poststimulation and wrapped the difference in a range between −0.5*π* and +0.5*π* (as phase shifts of −0.7*π* and + 0.3*π* functionally correspond; Supporting Information Figure S6). To compute the nonstimulated phase time series for every stimulation simulation, we ran a parallel simulation that was identical in all aspects except the stimulation intensity that was set to zero.

Group-level phase shifting portraits (Figure 4) were calculated for each parameter combination of stimulated region, phase, and FC state. We obtained the group-level phase shifting portraits by averaging the phase shifting portraits of individual stimulation simulations over the 25 samples of stimulation with the same region, phase, and FC state. Averages had to be performed on the complex plane as the quantities to be averaged are phase differences on the unit cycle.

#### State morphing: effective PRC.

To obtain the effective PRC of a given region (Figure 5C), we started by averaging the phase difference between stimulated and unstimulated simulations, in each individual stimulation simulation, over the last 140 steps window to discard initial transients induced by the stimulation pulse. Then, prior to averaging these phase shifts across samples we excluded samples where the classified FC state of the poststimulation window was not the same as the FC state at the time of stimulation. In this way, we could guarantee that the retained stimulation experiments were inducing only state morphing, and thus evaluate state-specific effective PRCs. Averages of phase shifts are performed once again on the complex plane. Since stimulating a region induces highly nonlocal phase shifting even in distant regions, we had to evaluate an individual effective PRC for each affected region in each parameter combination of phase, state, and region of stimulation. Error bars of the effective PRCs denote standard error of mean, normalized by the actual number of stimulation trials preserving the source FC state.

#### State switching.

To capture the probability difference of state switching due to stimulation, we first applied the *k-*nearest neighbor FC classifier previously trained (see FC states: classification section) in order to detect the poststimulation state the system lands into during the same poststimulation window used for PRC estimation (i.e., 140 timesteps poststimulation). We could thus estimate the probability of transiting from one state on another, using 25 samples (five windows, five cycles) for each parameter combination. As state switching can also occur spontaneously, we also computed this probability for the matching unstimulated simulation. We then subtracted the unstimulated from the stimulated probabilities of switching to obtain the probability difference of stimulation-induced state switching beyond possible spontaneous state switching in the unstimulated time series for each combination of phase-, region- and state-dependent measures (Figures 5A and 5D). For positive values of the probability difference the stimulation is inducing state switching, whereas negative values reflect a suppression of possibly natural state switching. Note that we perform stimulation in highly prototypical (and thus stable) windows, so that the unstimulated probabilities of switching were very small for Ψ_1_ and Ψ_3_ (and consistently at 0.2 for Ψ_2_).

#### Prediction of poststimulation phase shifting.

To predict stimulation-dependent phase shifting in the last window of the poststimulation timeseries, we use an RFR algorithm, implemented within the scikit-learn library (Version 0.21.3; Pedregosa et al., 2011). In such an approach, various decision trees are constructed in which the final leaves correspond to (discretized) predictions of the obtained phase shift and branches to follow are selected based on the values of the chosen input features. The final prediction is obtained averaging over a “forest” of many randomized trees. We used a maximal depth of five levels of branching for trees in the forest. We trained an independent RFR for each pair formed by a stimulated region (one of six possible: V1, DP, TEO, 2, 8B, STPr) and a region where effects were observed (one of 29 possible), that is, 168 classifiers to train (for each choice of the set of input features). Since the target output is a phase shift over the unit polar circle, but RFRs operate on the real axis, we transformed ΔΦ into a complex number exp^iΔΦ^ and used the real and imaginary part as the two dimensions of the output vector. Predictors included FC state-ignorant measures (phase of stimulation of the stimulated region; outgoing structural connectivity of the stimulated region SC_loc_) and FC state-aware measures (FC state label, PLV, PLV_local_, lag, lag_local_). The phase of stimulation was a 2 × 750 vector containing the real and imaginary phase of stimulation in radians at the stimulated region; the structural connectivity was a 29 × 750 vector consisting of the vector of outgoing structural connectivity of the stimulated region; the FC state was a 1 × 750 vector with the identity of the stimulated FC state identity; the PLV was a 406 × 750 vector containing the upper triangle of the PLV matrix in the corresponding stimulated window; PLV_local_ was a 8 (V1), 13 (TEO), 24 (STPr), 17 (DP), 16 (2), 18 (8B) × 750 vector containing the PLV between the stimulated region and the regions it structurally projected to; the lag was a 406 × 750 vector containing the upper triangle of the lag matrix in the corresponding stimulated window; Lag_local_ was a 8 (V1), 13 (TEO), 24 (STPr), 17 (DP), 16 (2), 18 (8B) × 750 vector containing the PLV between the stimulated region and the regions it structurally projected to. To explore how various parameter combinations affect the prediction accuracy, we concatenated the chosen predictors along the nonsample dimension so that we ended up with a *n* × 750 input vector. To make sure our results could generalize to novel data, we use tenfold cross-validation with a training size of 70%. Furthermore, we also stratified according to FC state, guaranteeing that each cross-validation fold included a balanced distribution of samples from each of the FC states, as classifiers may not be able to recognize classes that they have not been trained on.

## Acknowledgments

We thank Giovanni Rabuffo and Jan Fousek for the technical support in creating the model within The Virtual Brain software.

## Supporting Information

Supporting information for this article is available at https://doi.org/10.1162/NETN.a.548.

## Author Contributions

Sophie Benitez Stulz: Conceptualization; Data curation; Formal analysis; Investigation; Software; Visualization; Writing – original draft; Writing – review & editing. Samy Castro: Data curation; Investigation; Software; Validation; Writing – review & editing. Boris Gutkin: Conceptualization; Funding acquisition; Writing – review & editing. Matthieu Gilson: Supervision; Writing – original draft; Writing – review & editing. Demian Battaglia: Conceptualization; Funding acquisition; Methodology; Project administration; Supervision; Validation; Writing – original draft; Writing – review & editing.

## Funding Information

Demian A Battaglia, Association Nationale de la Recherche et de la Technologie (http://dx.doi.org/10.13039/501100003032), Award ID: ERMUNDY ANR-18-CE37-0014-02. Demian A Battaglia, Université de Strasbourg (http://dx.doi.org/10.13039/501100003768), Award ID: USIAS-2020-044.

## Data and Materials Availability Statement

All data needed to evaluate the conclusions in the paper are present in the paper and/or the Supplementary Materials. Code for simulation is available on request to the corresponding author and will be deposited in a public repository upon publication.

## Supplementary Material



## TECHNICAL TERMS

Transcranial magnetic stimulation (TMS): A magnetic field gun to stimulate a target brain region noninvasively (from outside the skull, no electrodes).
Dynamic functional connectivity (dFC): Spontaneous reconfiguration along time of networks describing patterns of correlation between the resting-state activity of different brain regions.
Functional connectivity (FC) state: A functional connectivity configuration transiently stable in time because of the system’s dynamics currently visiting a specific activity mode.
State switching: A more global modification of FC induced by a stimulation causing the system to transition to a different FC state.
State morphing: A slight local modification of FC that does not alter the FC state in which the stimulation is applied.
Phase response curve (PRC): A function measuring how the phase shifting effects of a pulse stimulation depend on the application phase of the perturbation.
Dynamic working point (DWP): Set by arousal in vivo and global parameters selection in silico, it determines which dynamical states the system can access.
State-aware vs. state-ignorant features: Prediction of perturbation effects can use features informative (aware) or not (ignorant) about which FC state the system currently is.
Effective phase response curve (PRC): The effective PRC measures phased stimulation effects on regions within a system, unlike real PRC defined for isolated nodes.
PING scenario: Scenario in which local oscillations are Generated from the Network interaction between coupled excitatory (Pyramidal) and Inhibitory local neuronal populations.
State-aware vs. state-ignorant features: Prediction of perturbation effects can use features informative (aware) or not (ignorant) about which FC state the system currently is.
