# Supplementary material for: Phase-dependent stimulation response is shaped by the brain’s dynamic functional connectivity
Source: Netw Neurosci. 2026 Apr 22;10(2):475–507. doi: 10.1162/NETN.a.548 (PMC13108500; doi:10.1162/NETN.a.548)
Supplement: Supplementary file 1 [file netn-10-2-475-s001.pdf]

Supplementary Figures for

Phase-dependent stimulation response is shaped by the brain's dynamic functional connectivity

Sophie Benitez Stulz[1], Samy Castro[2,3], Boris Gutkin[4], Matthieu Gilson[5 #], Demian Battaglia[2,3,1 # *]

[1]Aix-Marseille Université, INSERM, INS, Institut de Neurosciences des Systèmes (UMR 1106), 13005 Marseille, France

[2]Université de Strasbourg, CNRS, LNCA, Laboratoire de Neurosciences Cognitives et Adaptatives (UMR 7364), 67000 Strasbourg, France

[3] Université de Strasbourg, University of Strasbourg Institute for Advanced Studies (USIAS), 67000 Strasbourg, France

[4]Ecole Normale Supérieure - PSL University, LNC INSERM U960, DEC, 75005 Paris, France

[5]Aix-Marseille Université, CNRS, INT, Institut de Neurosciences de la Timone (UMR 7289), 13005 Marseille, France

# Shared last authorship; * Corresponding authorship: dbattaglia@unistra.fr

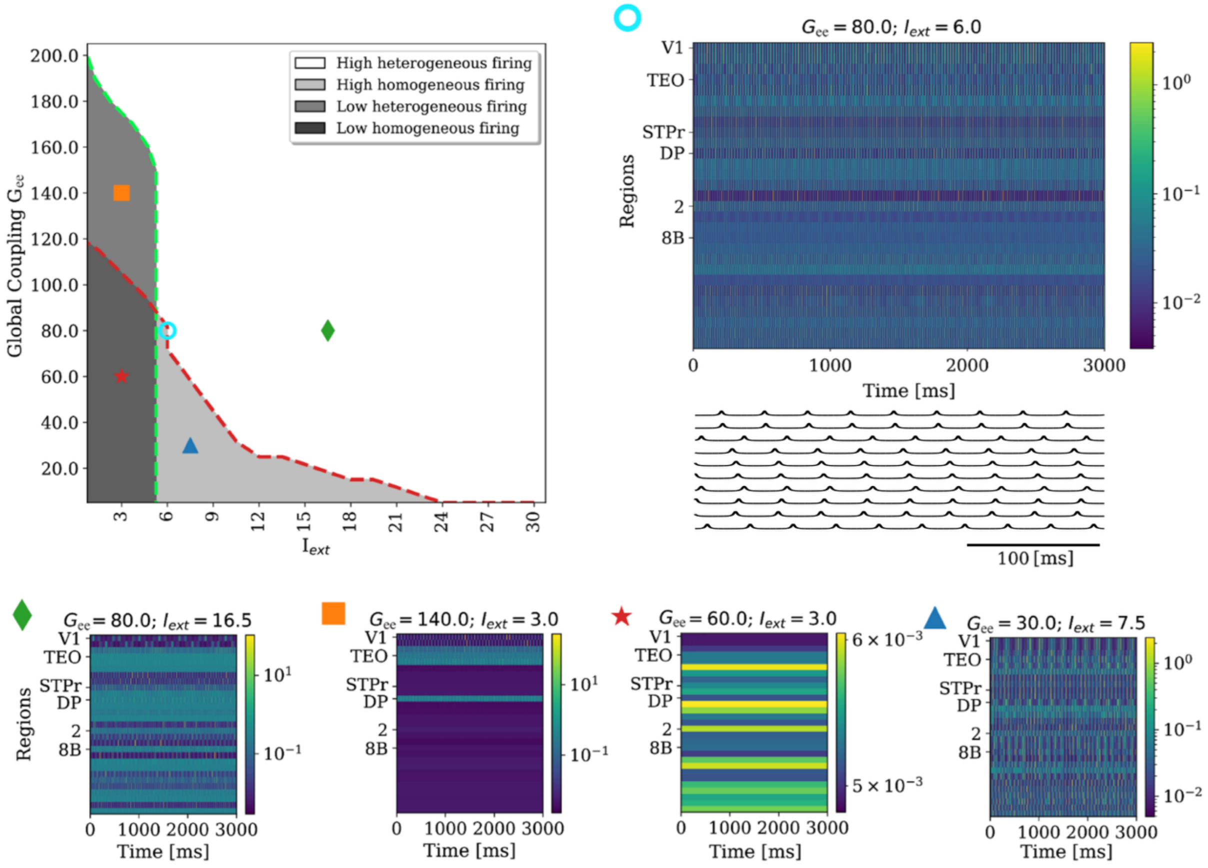

Figure S1. Example time series for different dynamical regimes. Top left: Reminder cartoon of the different dynamical regimes of the whole-brain model as a function of global parameters G_{ee} and I_{ext} (cf. Figure 2). Symbols mark example points for the four broad dynamical regimes: blue triangle (homogeneously low firing rate), orange square (homogeneously high firing rate), green rhombus (heterogeneously low firing rate), red star (heterogeneously high firing rate). The light blue circle represents the reference WP chosen for the analyses and stimulation experiments of Figures 3-6 (G_{ee}= 80.0, I_{ext} = 6.0). We display representative simulated time-series for the reference WP (top right; 3 seconds simulation, as well as representative time-series snippets zoomed over a shorter interval) and for each of the other four regime representative points (bottom, left to right). The labelled regions correspond to the regions which we individually select for stimulation in simulated experiments. Due to the deterministic simulation setup and the selected working point—chosen to maximize the likelihood of stable, stationary synchronization patterns—the resulting activity waveforms are regular, which facilitates phase extraction. However, they remain non-sinusoidal because of the intrinsic nonlinearities of the dynamical equations, directly derived from reduced spiking network models.

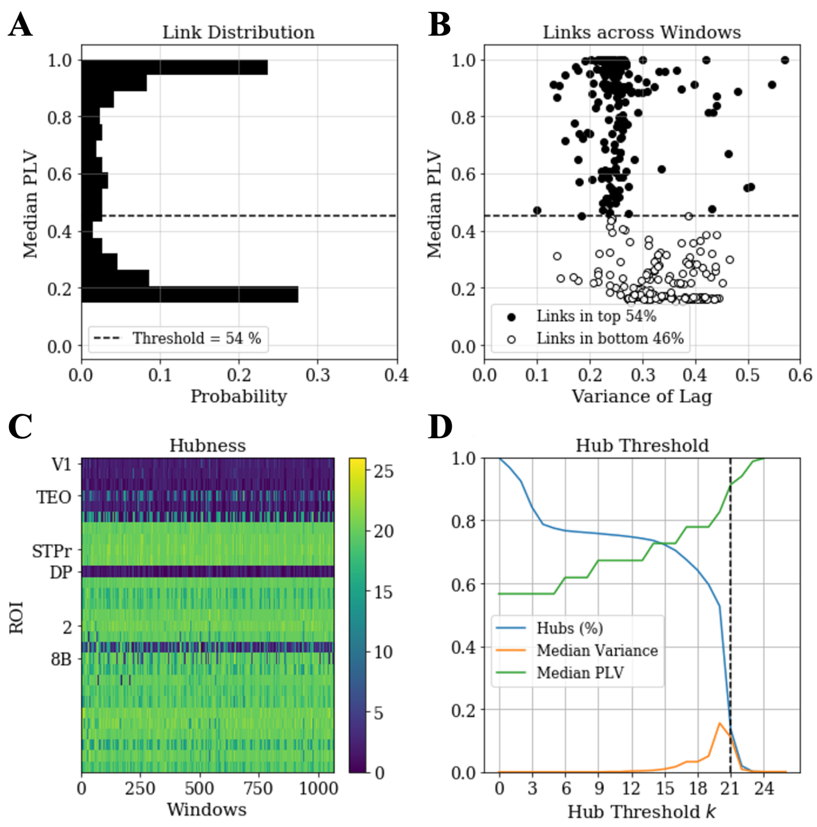

Figure S2. Pipeline for transient FC hub identification. Via a sliding window approach we generated temporal network of time-dependent PLV network frames, computed from simulated resting state time-series at the reference dynamic working point. Each parwise PLV link is thus time-dependent. **(A)** We show here the distribution of the median over time for each of the possible pairwise PLV links. This distribution is clearly bimodal which allows defining a natural threshold θ = 54% in the inter-peak gap to distinguish between strong and weak links. **(B)** Every link has an instantaneous lag, besides an instantaneous PLV strength value and this lag can also be variable. We show here a scatter plot of the median PLV vs the time-variance of phase lag (every dot corresponds to a different pairwise link). It is evident that some links strong on average also have a strong lag variability, corresponding to variability of phase-locking patterns. **(C)** We binarize each frame of the PLV temporal network, by retaining only PLV links transiently above the threshold θ. We show here the degree of each network node in each of the binarized PLV frames ("hubness"). **(D)** To decide whether a node is a hub or not we need to apply a further thresholding, this time not on the PLV strength of links, but on the degree of nodes. To choose a suitable threshold *k* defining the minimum node degree for a node to be considered a hub, we monitor three quantities as a function of growing *k*: the average fraction of hubs across frames (blue); the variance over time of being labeled as a hub or not (orange); and the median PLV level of nodes labeled as hubs with their neighbors. We then choose $k = 21$, which corresponds to simultaenous large phase synchronization of hubs with their neighbors, together with near maximal variability in

instantaneous hub appointment (many nodes are steadily appointed as hubs for lower thresholds, and almost never appointed as hubs for larger thresholds).

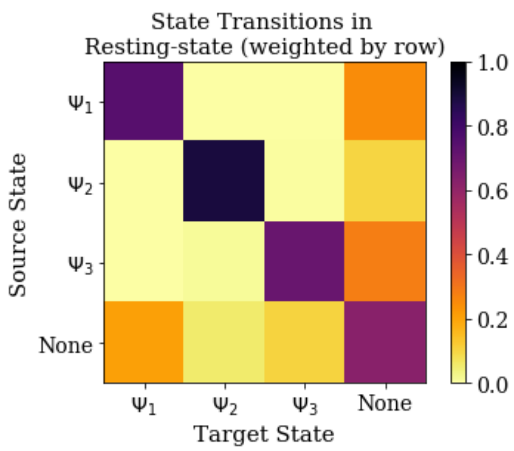

Figure S3. Intrinsic probability of state switching. In Fig. 3C we have identified three largely represented clusters of transient PLV/lag configurations and labelled them as FC states Ψ_1, Ψ_2 and Ψ_3. We add here a fourth label "None" grouping all transient FC configurations that do not belong to three reference clusters. Even in absence of stimulation, spontaneous transitions occur along spontaneous dynamics between these three (+1) states. We show here the probability that when the system visits state Ψ_i at a given time *t* it transits then to state Ψ_j at the consecutive time-step *t*+1. We evaluate this probability over simulations of unperturbed resting state dynamics at the chosen dynamic working point. This intrinsic probability of state switching is used to evaluate then the excess (or defect) probability of stimulation in a given state and a t a given phase to boost (or supress) state switching (cf. Figure 5D).

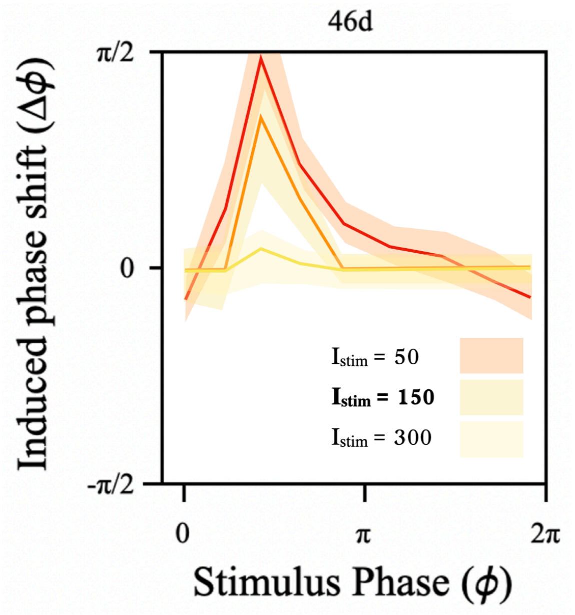

Figure S4. Dependency of phase response curves on stimulation intensity. We selected the stimulation intensity such that significant phase effects emerged only within specific phase ranges, and not uniformly across all phase bins. As an illustration, we show a PRC for region 46d, measured during stimulation of region 2 in state Ψ2, for three different stimulation intensities (with the bold value corresponding to the intensity used in Figures 4–6). With a smaller stimulation intensity, no phase bin exhibits significant phase shifting; with a larger intensity, significant phase shifts appear across all bins. Similar patterns are observed throughout the cortex, across different states and stimulated regions. The adopted stimulation intensity is therefore tuned to be neither too strong nor too weak (see Methods for the exact selection criteria).

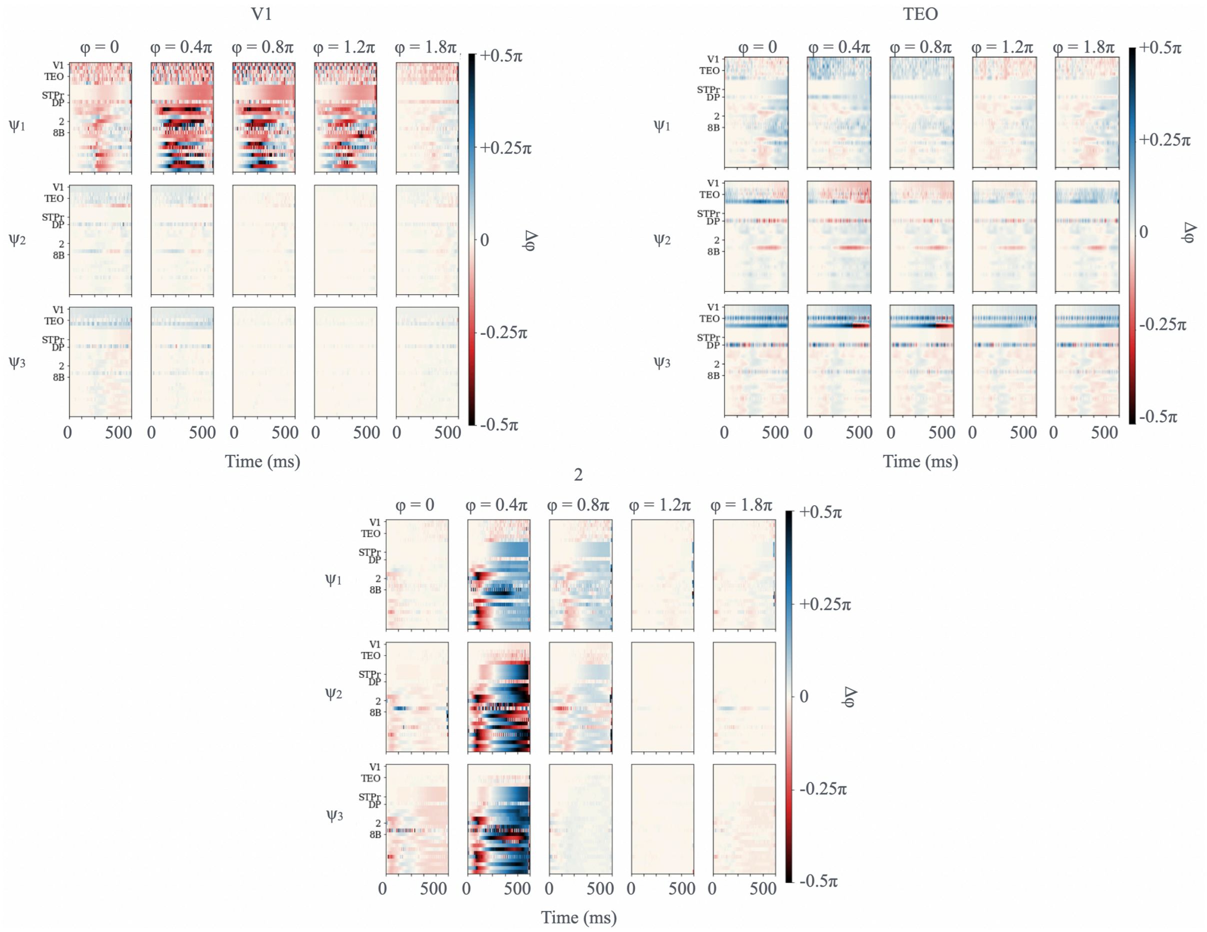

Figure S5. Phase shifting portraits for different stimulated regions. We show here phase shifting portraits obtained as in Figure 4 but presenting more systematic combinations of stimulated regions (V1, TEO or 2), FC states of stimulation (Ψ_1, Ψ_2 or Ψ_3) and stimulation phases (5 possible values). These portraits confirms the existence of strong region, state and phase dependencies of stimulation effects, which are nevertheless not completely unstructured.

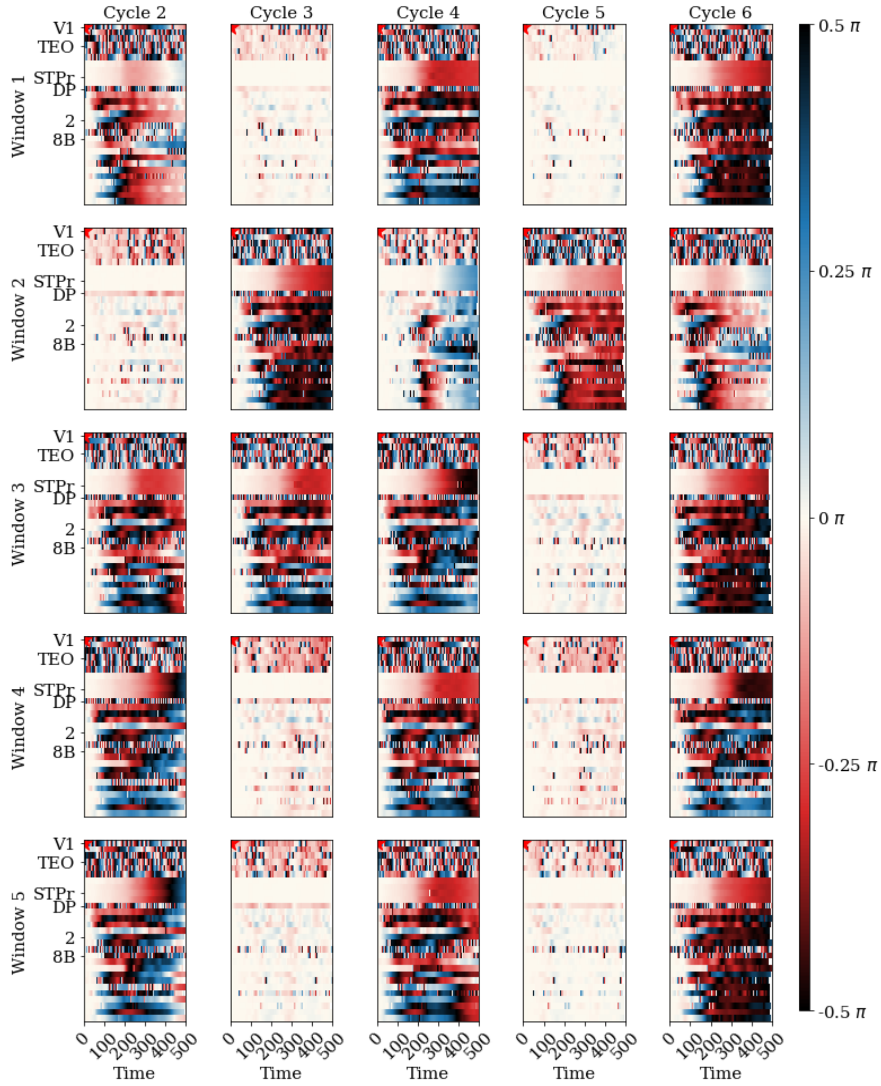

Figure S6. Example of trial-to-trial variability of stimulation effects. The phase shifting portraits shown in Figures 4 and S5 are averaged over many different realizations of pulse stimulation experiments with precisely the same parameter. For each fixed region, FC state and stimulation phase combination, we performed indeed stimulations in different FC state epochs (time-windows assigned to the state) and oscillation cycles inside the window (each window is long enough to accommodate multiple oscillation cycles). We show here an example of the variability of induced phase-shifting effects over windows and cycles for stimulation phase 0.2π, FC state Ψ_1 and region V1 (taking the five most typical windows within state Ψ_1). While many stimulation effects are reliably reproduced through the different repetitions, there is also variability unexplained by the three frozen parameters (region, state, phase; cf. the improvement in prediction by using full PLV and lag information rather than simple state label in Figure 6B).

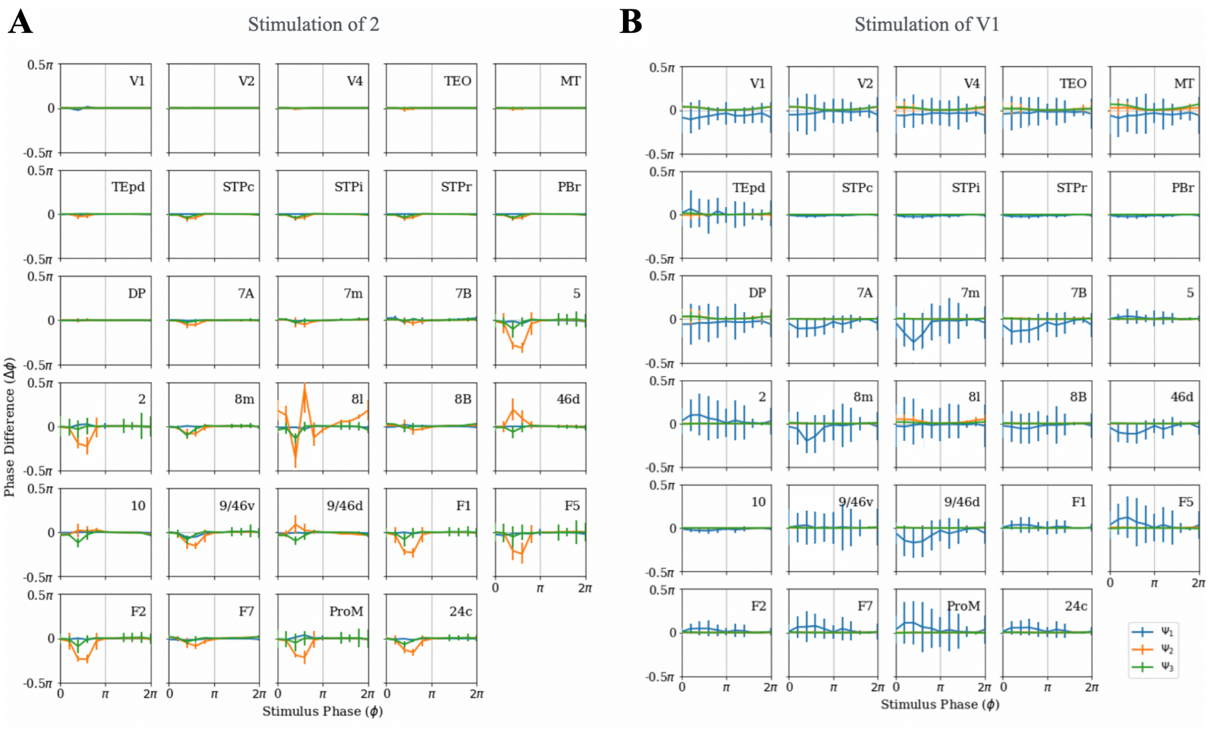

Figure S7. Effective PRCs under stimulation of different regions in different FC states. Effective PRCs describe state-morphing, giving the stimulation phase dependent phase-shift induced by phased stimulation not modifying the initial FC state prior stimulation. Phase-shifting effects are induced in a widespread manner, even in regions remote from the stimulated one. We give here effective PRCs for all 29 model regions when stimulating region 2 (**A**) or when stimulating region V1 (**B**) in each of the three reference states Ψ_1, Ψ_2 or Ψ_3. Stimulating region 2 leads to controllable phase morphing effects in state Ψ_2 in some frontal regions (e.g. region 2 itself, 46d, 9/46v…). Stimulating V1 leads to widespread phase-morphing effects in state Ψ_1 but less reliable and more unpredictable. Some regions have a very resilient oscillatory dynamics, hardly affected by state morphing irrespective of region and FC state (e.g. STPr, 8B, DP…). Lines indicate median, error bars s.e.m.

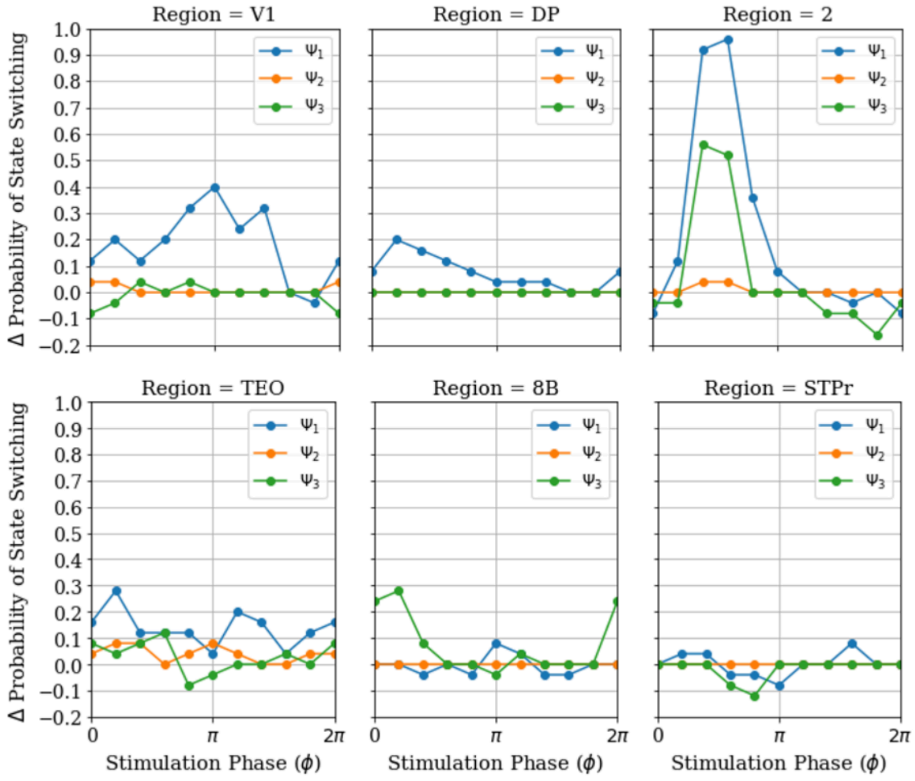

Figure S8. Probability of stimulation-induced state switching. As in Figure 5d, we show here the excess (or defect) probability of transiting to a different target FC state (relative to spontaneous intrinsic switching) induced by applying a phase stimulation, as a function of stimulated region, stimulation phase and stimulation FC state. The modulation of state switching probability when stimulating some regions (TEO, STPr, DP, 8B…) is smaller than when stimulating others. Strongest state-switching boosting effects are obtained for FC state Ψ_1 and Ψ3 when stimulating region 2 at phases 0.2π and 0.3π. We don't show shaded range for mean ± s.e.m. to avoid cluttering in the figure.

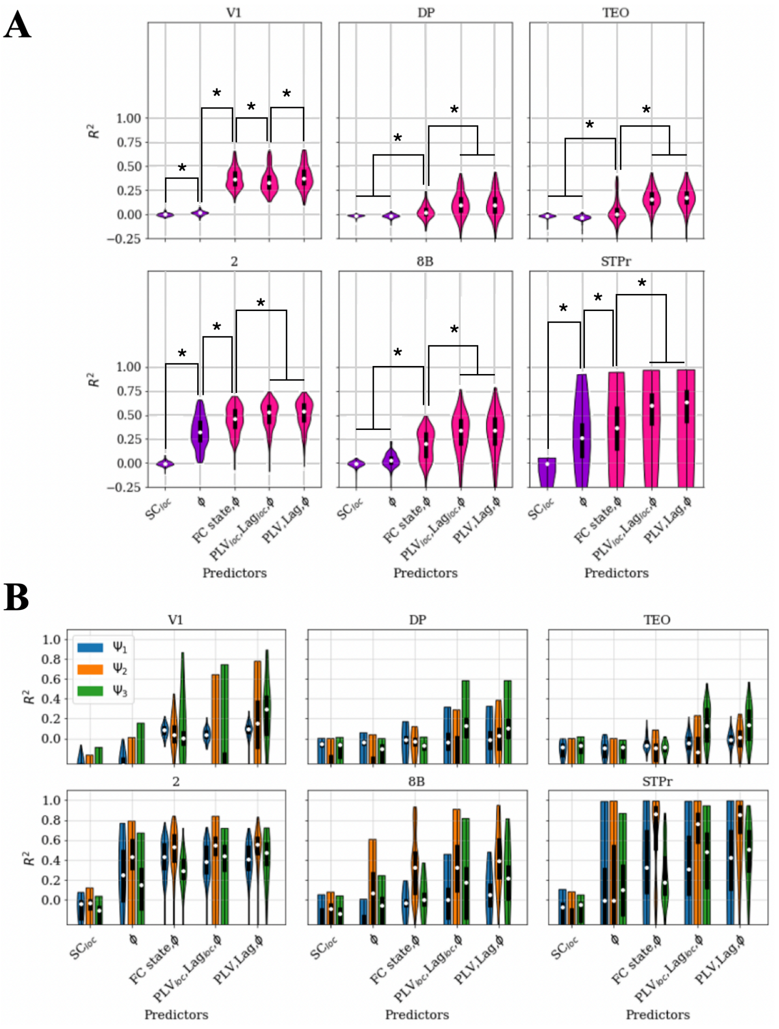

Figure S9. Performance of phase shifting prediction (additional information). (A) As in Figure 6, we show here 10-fold cross-validated performance (29 regions, so 290 points) of prediction using random forest regression fed with state-ignorant and state-aware feature sets (see Figure 6 for details). Beyond Figure 6, we show here performance for stimulation of different regions significance of all comparisons. **(B)** Same as panel A but performance is split by source state. The reduction in performance could not be attributed to the systematic underperformance of a single FC state but was spread over all FC states. In each violin plot the white dot marks the median and the thick black line marks the inner quartiles. Significance was tested with the Mann-Whitney U test and significance levels are indicated as follows: not drawn for $p \geq 0.05$; * for $p < 0.05$. Bonferroni correction for multiple comparisons was applied.
